# New terpenoids from the fermentation broth of the edible mushroom *Cyclocybe aegerita*

**DOI:** 10.3762/bjoc.15.98

**Published:** 2019-04-30

**Authors:** Frank Surup, Florian Hennicke, Nadine Sella, Maria Stroot, Steffen Bernecker, Sebastian Pfütze, Marc Stadler, Martin Rühl

**Affiliations:** 1 Microbial Drugs, Helmholtz Centre for Infection Research GmbH (HZI), Inhoffenstraße 7, 38124 Braunschweig, Germany; 2 German Centre for Infection Research Association (DZIF), Partner Site Hannover-Braunschweig, Inhoffenstraße 7, 38124 Braunschweig, Germany; 3 Junior Research Group Genetics and Genomics of Fungi, Senckenberg Gesellschaft für Naturforschung, Georg-Voigt-Str. 14–16, 60325 Frankfurt am Main, Germany; 4 Institute of Food Chemistry and Food Biotechnology, Justus Liebig University Giessen, Giessen, Germany; 5 Fraunhofer Institute for Molecular Biology and Applied Ecology IME Business Area Bioresources, Heinrich-Buff-Ring 17, 35392 Giessen, Germany

**Keywords:** bioinformatics, gene cluster analysis, natural products, secondary metabolites, structure elucidation, terpenes

## Abstract

The strophariaceous basidiomycete *Cyclocybe aegerita* (synonyms *Agrocybe aegerita* and *A. cylindracea*) is one of the most praised cultivated edible mushrooms and is being cultivated at large scale for food production. Furthermore, the fungus serves as a model organism to study fruiting body formation and the production of secondary metabolites during the life cycle of Basidiomycota. By studying the secondary metabolite profiles of *C. aegerita*, we found several terpenoids in submerged cultures. Aside from the main metabolite, bovistol (**1**), two new bovistol derivatives B and C (**2**, **3**) and pasteurestin C as a new protoilludane (**4**) were isolated by preparative HPLC. Their structures were elucidated by mass spectrometry and NMR spectroscopy. The relative configurations of **2**–**4** were assigned by ROESY correlations, and ^3^*J*_H,H_ coupling constants in the case of **4**. Applying quantitative PCR for gene expression validation, we linked the production of bovistol and its derivatives to the respective biosynthesis gene clusters.

## Introduction

The basidiomycete *Agrocybe aegerita* (synonym: *A. cylindracea*) was traditionally accommodated in the genus *Agrocybe* (family Bolbitiaceae) until a recent phylogenetic study based on comparisons of rDNA sequence data has resulted in its placement in the Strophariaceae family and it was accordingly moved into the resurrected genus *Cyclocybe* Velenovsky [1–2]. The original publication by Vizzini et al. [1] was published in a regional journal, rather than in one of the leading peer-reviewed taxonomic journals, and the authors did not follow good scientific practice for typification when proposing these taxonomic changes. However, they subsequently published their entries in Index Fungorum, making them valid according to the current nomenclature rules, and the phylogenetic tree they presented clearly revealed that the species now accommodated in the genus *Cyclocybe* are more closely related to the Strophariaceae and phylogenetically rather distinct from those of the family Bolbitiaceae, including the type species of *Agrocybe* which is *A. praecox*. Therefore, the currently valid scientific name of the fungus is *Cyclocybe aegerita* (V. Brig.) Vizzini.

**Figure 1:**
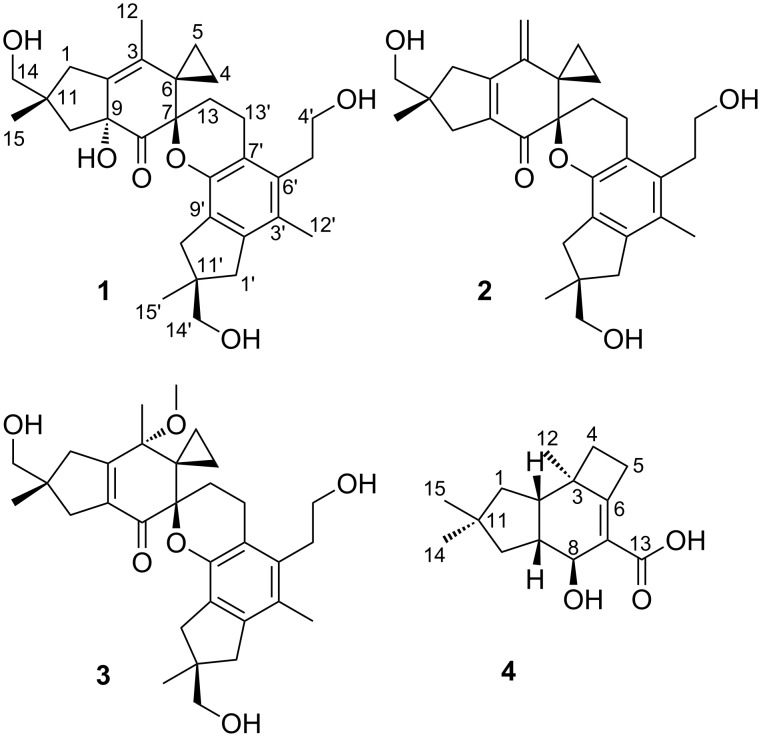
Structures of the isolated metabolites bovistol A (**1**), its new derivatives bovistol B (**2**) and C (**3**), as well as the new protoilludane pasteurestin C (**4**).

In fact, *C. aegerita* is a rather important fungal species with regard to practical applications, as i) it belongs to the edible mushrooms that are being cultivated at industrial scale and it is highly esteemed for its excellent aroma and ii) it has been used as a model organism for microbiological and genetic investigations on fruiting body formation for many years. Herzog et al. [3] have recently reported a parental dikaryotic strain *C. aegerita* AAE-3 that completes its life cycle on agar plates in only three weeks, along with a set of sibling monokaryons derived from it. Among these monokaryons, *C. aegerita* AAE-3-32 has been used for histological analysis of monokaryotic fruiting sensu stricto (mushroom formation without previous mating) and, together with *C. aegerita* AAE-3-13, for exploring molecular tools for transformation and gene of interest expression, which has just been published [4]. These strains could serve well for studies exploring the factors regulating monokaryotic fruiting in comparison to dikaryotic mushroom formation. In addition, strains of this fungal species show a reliable growth behaviour in liquid culture and could eventually serve as hosts for heterologous production of secondary metabolites derived from other Basidiomycota that are more difficult or even impossible to culture. With these goals in mind, we have initiated extensive studies of the secondary metabolism of the aforementioned strains, targeting both volatile and non-volatile compounds. The present paper will describe the discovery of one known and three new non-volatile terpenoids (Figure 1) that were isolated from liquid cultures of *C. aegerita* and their physicochemical and preliminary biological characterisation.

## Results and Discussion

Both the ethyl acetate extract of the culture filtrate and the acetone extract of the mycelium of *C. aegerita*, grown in ZM/2 medium, contained a major metabolite with a molecular mass of 496 Da, as detected by HPLC–MS analysis. Its molecular formula C_30_H_40_O_6_ was deduced from its [M + Na]^+^ peak at *m/z* 519.2718 in the HRESIMS spectrum. ^1^H and ^1^H,^13^C-HSQC NMR data (Table 1) indicated the presence of four methyls and twelve methylenes, three of them oxomethylene groups. A database search with this data within the Chapman & Hall Dictionary of Natural Products on DVD suggested its identity as bovistol, which was confirmed by the elucidation of the structure by COSY and HMBC NMR data [5].

**Table 1:** NMR shifts (^1^H 700 MHz, ^13^C 175 MHz) of bovistol A–C (**1**–**3**) in chloroform-*d*.

	**1**		**2**		**3**	
Atom#	^13^C, mult.	^1^H, mult. (*J*, Hz)	^13^C, mult.	^1^H, mult. (*J*, Hz)	^13^C, mult.	^1^H, mult. (*J*, Hz)

1	38.8, CH_2_	2.45, d (15.4)/2.40, d (15.4)	40.0, CH_2_	2.32, m/2.70, m	43.3, CH_2_	2.75, dt (18.1, 2.0)/2.43, br d (18.1)
2	135.4, C		156.0, C		161.1, C	
3	130.7, C		143.6, C		75.4, C	
4	7.5, CH_2_	1.26, m/0.64, ddd (9.8, 5.9, 5.7)	5.2, CH_2_	1.05, m/0.87, m	4.0, CH_2_	1.11, ddd (9.3, 6.7, 4.8)/0.30, ddd (9.3, 6.6, 4.8)
5	8.6, CH_2_	0.89, dt (10.0, 5.7)/0.79, dt (10.0, 5.9)	13.1, CH_2_	1.46, m/0.29, m	5.7, CH_2_	0.88, m
6	32.4, C		30.9, C		30.0, C	
7	80.9, C		79.3, C		79.7, C	
8	207.6, C		196.7, C		197.2, C	
9	80.8, C		134.2, C		134.2, C	
10	44.9, CH_2_	1.94, d (14.2)/1.48, d (14.2)	43.1, CH_2_	2.75, m/2.55, m	39.5, CH_2_	2.58, dt (16.8, 2.0)/2.24, br d (16.8)
11	41.2, C		42.8, C		42.5, C	
12	15.1, CH_3_	1.50, s	112.3, CH_2_	5.16, s/5.11, s	18.5, CH_3_	1.164, s
13	27.7, CH_2_	2.84, m/1.72, td (13.0, 5.8)	29.2, CH_2_	2.21, m/1.89, td (13.5, 5.0)	28.5, CH_2_	2.78, m/1.72, td (13.5, 5.0)
14	71.6, CH_2_	3.26, s	70.3, CH_2_	3.49, s	70.4, CH_2_	3.48, s
15	26.4, CH_3_	1.26, s	24.8, CH_3_	1.20, s	24.7, CH_3_	1.160, s
1’	43.0, CH_2_	2.86, d (16.0)/2.61, d (16.0)	42.8, CH_2_	2.86, m/2.60, m	42.8, CH_2_	2.84, d (16.0)/2.59, d (16.0)
2’	141.6, C		141.4, C		141.1, C	
3’	124.5, C		124.2, C		124.1, C	
4’	61.7, CH_2_	3.69, m	62.0, CH_2_	3.70, br t (6.8)	62.1, CH_2_	3.71, m
5’	31.9, CH_2_	2.83, m	32.2, CH_2_	2.86, m	32.1, CH_2_	2.88, m
6’	133.0, C		132.4, C		132.2, C	
7’	118.6, C		118.4, C		119.1, C	
8’	148.5, C		149.2, C		149.1, C	
9’	127.2, C		126.8, C		126.6, C	
10’	39.4, CH_2_	2.94, d (16.4)/2.55, d (16.4)	39.6, CH_2_	2.86, m/2.52, m	39.6, CH_2_	2.83, d (16.5)/2.51, d (16.5)
11’	43.8, C		43.9, C		43.9, C	
12’	15.6, CH_3_	2.12, s	15.5, CH_3_	2.14, s	15.5, CH_3_	2.14, s
13’	21.2, CH_2_	2.72, m	20.1, CH_2_	2.40, m	19.9, CH_2_	2.78, m/2.40, m
14’	71.3, CH_2_	3.50, s	71.2, CH_2_	3.51, s	71.2, CH_2_	3.51, s
15’	25.1, CH_3_	1.19, s	24.9, CH_3_	1.21, s	24.9, CH_3_	1.20, s
3OMe					50.5, CH_3_	3.33, s

In the course of the isolation of **1** the minor metabolites **2** and **3** accrued. Metabolite **2** was analysed for a molecular weight of 478 Da. Its molecular formula C_30_H_38_O_5_, deduced from HRESIMS data, indicated the formal loss of one molecule of water. ^1^H and HSQC NMR data (Table 1) of **2** were very similar to those of **1**, with the exception of the replacement of methyl CH_3_-12 by an exomethylene group. HMBC correlations from both exo-methylene protons 12-H_2_ to C-2, C-3, C-4, in addition to those from both 1-H_2_ and 10-H_2_ to the olefinic carbons C-2 and C-9, confirmed the structure of **2** (Figure 1). For **3**, HRESIMS data revealed its molecular formula as C_31_H_42_O_6_. The ^1^H and ^13^C NMR data (Table 1) were highly similar to those of **1**, with the key difference being an additional methoxy group (δ_H_ 3.33/δ_C_ 50.5). This methoxy was connected to C-3 due to its HMBC correlation to this carbon atom, along to the ones from 1-H_2_, 10-H_2_ and 12-H_3_ to C-3. Compared to **1**, the Δ^2,3^ double bond is shifted to Δ^2,9^, explaining the high field shift of the conjugated ketone C-8 (δ_C_ 197.2). A ROESY correlation between 3-OCH_3_ and 13-H_a_ revealed a 3*S* configuration. The closest known structural relative of **3** is the 3-demethoxy-3-hydroxy derivative of **3**, which has been described as a spontaneous dimerization product of psathyrellon B [5].

In addition to disesquiterpenoids **1**–**3**, metabolite **4** with a molecular mass of 250 Da was isolated. ^1^H and ^1^H,^13^C-HSQC NMR data (Table 2) revealed the presence of three methyls, four methylenes and three methines, one of them oxygenated. The ^13^C NMR spectrum indicated furthermore a carboxylic acid in addition to two olefinic and two aliphatic carbons devoid bound protons. A large spin system comprising 1-H_2_/2-H/9-H(10-H_2_)/8-H was constructed by COSY and TOCSY correlations in addition to the small one of 4-H_2_/5-H_2_. These spin systems were connected by HMBC correlations to form the protoilludane skeleton, mainly to note the correlations from 14-H_3_ and 15-H_3_ to 1-H_2_/10-H_2_, 12-H_3_ to C-2/C-3/C-4/C-6, 5-H_2_ to C-6/C-7 and 8-H to C-6/C-7/C-13. The strong ROESY correlation between 2-H and 9-H indicated a *cis* configuration between these protons, and the large coupling constant between 8-H and 9-H, observed in the signal of 8-H, a *trans* configuration of 8-H/9-H. Finally, the ROESY correlation between 12-H_3_ and 1-H_b_ indicated methyl C-12 being on the opposite site of the molecular plane as 2-H and 9-H. This assignment was confirmed by the comparison of the ^13^C NMR shifts to pasteurestin A and B [6–7]. Since the absolute stereochemistry has been demonstrated for pasteurestins A and B by total synthesis, we tentatively conclude a 2*S*,3*R*,8*S*,9*R* absolute configuration for pasteurestin C (**4**). The systematic name for **4** is (4*S*,4a*R*,7a*S*,7b*R*)-4-hydroxy-6,6,7b-trimethyl-2,4,4a,5,6,7,7a,7b-octahydro-1*H*-cyclobuta[*e*]indene-3-carboxylic acid.

**Table 2:** NMR data (^1^H 700 MHz, ^13^C 175 MHz) of compound **4** in acetone-*d*_6_.

Atom#	C Shift	H Shift	COSY	HMBC

1	41.6, CH_2_	1.44, m	1, 2	14, 11, 10, 9
		1.39, m	1, 2	14, 15, 11, 2, 3
2	45.9, CH	2.43, m	1, 1	9, 12, 4, 3, 9, 8
3	47.8, C			
4	36.4, CH_2_	1.95, m	5, 5	12, 5, 2, 3, 7, 6
5	29.9, CH_2_	3.13, m	4, 5, 8	4, 3, 7, 6
		3.02, m	4, 5	3, 7, 6
6	170.4, C			
7	122.1, C			
8	72.3, CH	4.27, dt (8.0, 2.0)	9, 5	10, 7, 13, 6
9	50.9, CH	2.40, m	10, 10, 8	2, 3, 8
10	47.4, CH_2_	1.12, m	10, 9	14, 15, 11, 9, 8
		1.793, br dd (11.2, 7.5)	10, 9	14, 1, 2
11	40.4, C			
12	20.3, CH_3_	1.14, m		4, 2, 3, 6
13	167.8, C			
14	27.4, CH_3_	0.96, s		15, 11, 1, 10
15	29.8, CH_3_	1.09, m		14, 11, 1, 10

Bovistol A (**1**) showed weak cytotoxic effects (IC_50_ for L929 = 15 µg/mL, for KB3.1 = 7 µg/mL), but was inactive against all test organisms in our standard test panel, comprising selected Gram-positive and Gram-negative bacteria as well as fungi [8]. Compound **4** was inactive in all assays of our test panel, and **3** could not be tested due to the insufficient amount isolated.

Our finding of the production of **1**–**4** by *C. aegerita* expands the number of secondary metabolites known from this fungus. From fungal cultures of the genus *Cyclocybe* the production of a broad variety of metabolites is known. This includes polyacetylenes [9–10] as well as sesquiterpenoids with illudine [11], aromadendrane [12], marasmene [13] and fomannosane [14] type skeletons. Although bovistol could formally be supposed to be a triterpene, it is thought to be derived by a hetero-Diels–Alder reaction of two sesquiterpenes to form a dimeric sesquiterpenoid [15].

**Figure 2:**
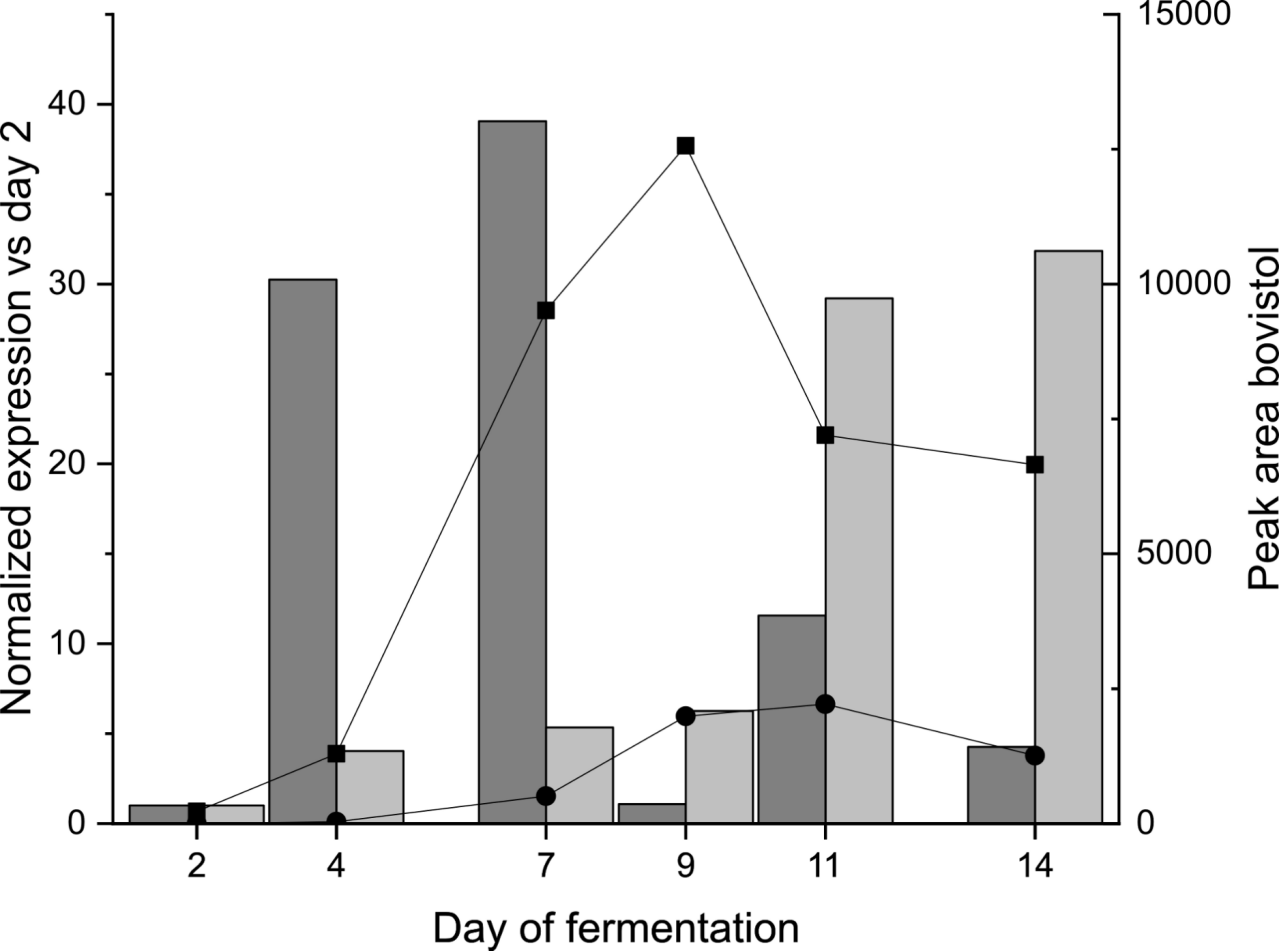
Relative normalized expression of the putative sesquiterpene synthase genes going by the gene IDs AAE3_04120 (dark grey) and AAE3_10454 (light grey) from the *C. aegerita* AAE-3 genome ([16], http://www.thines-ab.senckenberg.de/agrocybe_genome) compared to the peak areas of **1** in the supernatant (black squares) and in the fungal mycelium (black circles).

In the recently published genome of *C. aegerita* [16], two putative sesquiterpene synthase gene clusters have been identified on the basis of the published Δ^6^-protoilludene gene cluster of *Omphalotus olearius* [17]. The protein sequences of the genes clustering adjacent to the putative sesquiterpene synthase genes going by the gene IDs AAE3_04120 and AAE3_10454 (http://www.thines-lab.senckenberg.de/agrocybe_genome) reveal the presence of P450 monooxygenases, oxidoreductases as well as one putative Diels-Alderase 1 kb downstream of the putative Δ^6^-protoilludene synthase gene going by the gene ID AAE3_04120. To determine the correspondence between both putative sesquiterpene synthases and the analysed secondary metabolites **1**–**4** *C. aegerita* AAE-3 was cultivated in a stirred vessel bioreactor. Mycelial samples were analysed for the presence of the gene transcripts and **1** and **3** (Figure 2). At the beginning of the fermentation, the putative sesquiterpene synthase gene with the gene ID AAE3_04120 was upregulated with a maximum of transcripts at day 7 of cultivation. The second gene with the gene ID AAE3_10454 showed a more slight increase expression peaking at day 11 and day 14. The peak area of **1** in the supernatant increased until day 9 of cultivation and lowered afterwards, whereas in the mycelium the bovistol peak area increased until day 11 and dropped afterwards. This steady increase of **1** until day 9 respectively day 11 resembles the preceding transcriptional upregulation of the expression of the gene with the ID AAE3_04120 which indicates that this gene is presumably involved in the bovistol synthesis pathway. In addition, the deduced protein sequence of the adjacent gene with the ID AAE3_04121 shows similarities to the Diels-Alderase Sol5 from *Alternaria solani*, which is involved in the cycloaddition of prosolanapyrone II into solanapyrone [18]. A similar cycloaddition is needed to form bovistol out of two illudine precursors. Although the respective illudine has not been detected in this study, it is known that *C. aegerita* is able to produce several illudanes [11]. As proposed in recent studies on sesquiterpenes in *O. olearius* [17], *Stereum hirsutum* [19] and *Diaporthe* sp. [20] the illudin precursor Δ^6^-protoilludene undergoes subsequent reactions catalyzed by enzymes, whose genes cluster with the Δ^6^-protoilludene synthase. Nevertheless, evidence is still missing. In *C. aegerita*, Δ^6^-protoilludene is seemingly provided by the putative sesquiterpene synthase AAE3_04120. Combining these indications, an enzymatic cascade coded by the AAE3_04120 cluster is transforming Δ^6^-protoilludene into illudine(s) and the corresponding bovistol in *C. aegerita* (Figure S2, Supporting Information File 1). Further research has to verify this assumption.

## Conclusion

We identified bovistol A (**1**) as the main metabolite of *C. aegerita* in cultures. In parallel, we isolated its new disesquiterpenoid derivatives bovistol B (**2**) and C (**3**) as well as the new protoilludane pasteurestin C (**4**). By a qPCR approach, we were able to link the production of bovistol to the putative Δ^6^-protoilludene synthase AAE3_04120 whose gene is located adjacent to a Diels-Alderase needed for cycloaddition of two illudine monomers. With this information, we made a biosynthesis proposal for these metabolites. Further studies will address this assumption to prove its validity.

## Experimental

### General

Optical rotations were measured on a Perkin-Elmer 241 spectrometer, the UV spectra on a Shimadzu UV–vis spectrophotometer UV-2450. NMR spectra were recorded with a Bruker Avance III 700 spectrometer, equipped with 5 mm TCI cryoprobe (^1^H 700 MHz, ^13^C 175 MHz). Chemical shifts δ were referenced to the solvents chloroform-*d* (^1^H, δ = 7.27 ppm; ^13^C, δ = 77.0 ppm), acetone-*d*_6_ (^1^H, δ = 2.05 ppm; ^13^C, δ = 29.92 ppm). ESIMS spectra were acquired on an Amazon ion trap mass spectrometer (Bruker Daltonik); HRESIMS spectra were acquired on a Maxis time-of-flight mass spectrometer (Bruker Daltonik), both combined with an Agilent 1200 series HPLC-UV system [column 2.1 × 50 mm, 1.7 μm, C18 Acuity UPLC BEH (Waters), solvent A: H_2_O + 0.1% formic acid; solvent B: ACN + 0.1% formic acid, gradient: 5% B for 0.5 min increasing to 100% B in 19.5 min, maintaining 100% B for 5 min, flow rate = 0.6 mL min^−1^, UV detection at 200–600 nm].

### Fermentation in shaking flasks

A culture of the strain *C. aegerita* AAE-3 with a total volume of 4 L was prepared in ZM ½ medium [molasses 47% (Nord Zucker AG Schladen, Germany) 5.00 g/L, oatmeal (Herrnmühle, Harald Feick OHG, Reichelsheim, Germany) 5.00 g/L, D(+)-sucrose (Carl Roth GmbH & Co, Karlsruhe, Germany) 4.00 g/L, D-mannitol (AppliChem GmbH, ITW Company, Darmstadt, Germany) 4.00 g/L, D-glucose monohydrate (Cargill Holding Germany GmbH, Krefeld, Germany) 1.50 g/L, CaCO_3_ (Carl Roth GmbH & Co KG, Karlsruhe, Germany) 1.50 g/L, lactalbumin hydrolysate (Oxoid LDT, Basingstocke, Hampshire, England) 0.50 g/L, (NH_4_)_2_SO_4_ 0.50 g/L]. The pH value of the medium was set to 7.2. To inoculate the medium, 5 mL of a preculture were used. The inoculated shaking flasks were incubated on a lab shaker (NORD Drive systems SK CSX-3H, Getriebebau NORD GmbH & Co KG Drive Systems) at 23 °C. After one week of incubation the glucose concentration in the medium was tested daily. If no more free glucose could be found in the medium, the cultivation was extracted.

### Extraction

The mycelium was separated from the fermentation broth with the help of a fluted filter. The filtered mycelium was overlaid with actone (1:1 ratio) and digested in an ultrasonic bath (Sonorex digital 10P, BANDELIN electronic GmbH & Co KG, Berlin, Germany) for 30 min (two times). After the digest the mycelium was separated from the filtrate. The filtrate was narrowed up to the aqueous phase in vacuo. The aqueous phase was shaken out in a 1:1 ratio with ethyl acetate (+ 50 mL deionised H_2_O) in a separating funnel (two times) and dried with water-free sodium sulfate (Na_2_SO_4_, ACROS Organics, New Jersey, USA). The sodium sulfate was filtered off and the extract was narrowed up to the dry with a rotary evaporator. The crude extract was dissolved in 4 mL methanol, evaporated under a nitrogen evaporator (VLM GmbH, Bielefeld, Germany) and stored in a freezer at −20 °C. The crude extract of the fermentation broth was obtained the same way.

### Isolation

The crude extract obtained from the extraction was dissolved in 1 mL of methanol and further separated via RP-LC with deionized water (+ 0.05% TFA, solvent A) and methanol (+ 0.05%, solvent B) by a Gilson RP-HPLC system (Middleton, Wisconsin, USA) equipped with a GX-271 Liquid handler, a diode array detector (DAD) 172 and a 305 and 306 pump. The separation was performed with a VP Nucleodur C18ec (150 × 40 mm, 7 µm; Macherey-Nagel, Düren, Germany) column and a flow rate of 20 mL/min. The gradient was set from 30 to 70% of solvent B in 45 min, with an increase to 100% B in 15 min, followed by isocratic conditions at 100% B for 15 min. All LC fractions were collected according to the UV absorption at 210 nm. Methanol was evaporated in vacuo. The aqueous residues were frozen and then removed by using an Alpha 1-4 LSC freeze dryer (Christ, Osterode, Germany). 12.6 mg of fraction V were obtained as a mixture of compounds **1**, **2** and **3**. Furthermore fraction VI yielded 5.2 mg of pure compound **4**.

Subsequently fraction V was separated via another RP-LC under different conditions. The separation was performed with the same Gilson RP-HPLC system and flow by using a Gemini 10u C18 (250 × 21.20 mm, 10 micron, Phenomenex Inc., Torrance, USA) column. Solvent B was changed to acetonitrile and also no TFA was added. The gradient was set from 50% to 75% of solvent B in 45 min with an increase to 100% B in 15 min, followed by isocratic conditions at 100% B for 15 min. Collection and work-up of the obtained fractions was performed as described above. This separation yielded 4 mg of compound **1**, 2.2 mg of compound **2** and 0.4 mg of compound **3**.

**Bovistol A (1):** Colourless oil; ^1^H and ^13^C NMR data (^1^H 700 MHz, ^13^C 175 MHz) in CDCl_3_: see Table 1; ESIMS (*m/z*): 1015.52 [2M + Na]^+^, 479.30 [M + H − H_2_O]^+^, 991.64 [2M − H]^−^, 541.39 [M + HCOO]^−^; HRESIMS (*m/z*): [M + Na]^+^ calcd for C_30_H_40_O_6_Na, 519.2717; found, 519.2718.

**Bovistol B (2):** colourless oil; [α]_D_^25^ +3 (*c* 0.1, CH_3_OH); ^1^H and ^13^C NMR data (^1^H 700 MHz, ^13^C 175 MHz) in CDCl_3_: see Table 1; ESIMS (*m/z*): 979.57 [2M + Na]^+^, 957.61 [2M + H]^+^, 479.35 [M + H]^+^, 523.30 [M + HCOO]^−^, 477.29 [M − H]^−^; HRESIMS (*m/z*): [M + Na]^+^ calcd for C_30_H_38_O_5_Na, 501.2585; found, 501.2606.

**Bovistol C (3):** Colourless oil; [α]_D_^25^ +106 (*c* 0.1, CH_3_OH); ^1^H and ^13^C NMR data (^1^H 700 MHz, ^13^C 175 MHz) in CDCl_3_: see Table 1; ESIMS (*m/z*): 1043.65 [2M + Na]^+^, 533.37 [M + Na]^+^, 493.31 [M + H − H_2_O]^+^, 509.30 [M − H]^−^, 555.32 [M + HCOO]^−^; HRESIMS (*m/z*): [M + Na]^+^ calcd for C_31_H_42_O_6_Na, 533.2874; found, 533.2859; [M + H]^+^ calcd for C_31_H_43_O_6_, 511.3054; found, 511.3044.

**Pasteurestin C (4):** Colourless oil; [α]_D_^25^ +21 (*c* 0.1, CH_3_OH); ^1^H and ^13^C NMR data (^1^H 700 MHz, ^13^C 175 MHz) in acetone-*d*_6_: see Table 2; ESIMS (*m/z*): 523.32 [2M + Na]^+^, 233.13 [M + H − H_2_O]^+^, 249.04 [M − H]^−^; HRESIMS (*m/z*): [M + Na]^+^ calcd for C_15_H_22_O_3_Na, 273.1461; found, 273.1460; [M + H]^+^ calcd for C_15_H_23_O_3_, 251.1642; found, 251.1637.

### Fermentation in 10 L scale bioreactor

A seed culture of the strain *C. aegerita* AAE-3 with a total volume of 500 mL was prepared in ZM ½ medium. After incubation for 11 days the seed culture was homogenized with an ULTRA-TURRAX under sterile conditions and used for inoculation of a 15 L bioreactor (xCUBIO in-situ bbi biotech) filled with 10 L ZM ½ medium. The pH value was set to 7.2 (unregulated); the DO was also not regulated. The temperature was regulated at 23 °C. Furthermore the submerged aeration rate and stirrer speed was fixed to 0.15 vvm and 200 rpm (rushton turbine). For foam destruction into the process Tego Antifoam D2310 (Evonik Nutrition & Care GmbH) was used. After 29 days of cultivation the fermentation broth was harvested. The biomass and suspended substrates was separated by centrifugation.

### RNA extraction, cDNA synthesis and qPCR

During fermentation, mycelial samples were taken at day 2, 4, 7, 9, 11 and 14 and stored in RNAlater (Qiagen, Venlo, Netherlands) until further use. Fungal mycelium was freeze-dried and ground with liquid nitrogen. RNA was extracted from ground mycelium using Ambion TRIzol™ Reagent (life Technologies, Carlsberg, California, USA) according to the manufacturer’s instructions with minor changes according to the method of Chomczynski and Sacchi [21]. RNA concentration was determined photometrically by a NanoPhotometer^®^ Pearl (Implen, Munich, Germany). Reverse transcription was performed with the Invitrogen M-MLV Reverse Transcriptase kit (ThermoFisher Scientific, Waltham, Massachusetts, USA) according to the manufacturer’s protocol. 10 µL of extracted RNA and 1 µL of 10 µM oligo-(dT)_30_ primer (Eurofins, Waltham, Massachusetts, USA) were used for cDNA synthesis. For removal of RNA in the transcribed cDNA sample, 1 µL of AMRESCO RNase A (VWR International, Radnor, Pennsylvania, USA) was added and the mixture was incubated at 37 °C for 20 min. Primers for qPCR analysis were designed using Geneious 11.0.4. (Biomatters, Auckland, New Zealand). Primer pairs for *C. aegerita* housekeeping genes going by the gene IDs AAE3_02268 and AAE3_07669 (http://www.thines-lab.senckenberg.de/agrocybe_genome) have been identified and validated by NormFinder and geNorm algorithm to be the best combination for qPCR-based transcription analyses of *C. aegerita* by means of qPCR (data not published). Briefly, KAPA SYBR^®^ FAST qPCR Master Mix (Kapa Biosystems, Wilmington, MA, USA), 900 nM forward primer, 900 nM reverse Primer (Table 3), 10 ng of cDNA and nuclease-free water were mixed. The qPCR reactions were performed in triplicates using the CFX Connect™ RT-PCR Detection System (Bio-Rad Laboratories, Hercules, CA, USA). The following conditions were applied: enzyme activation at 94 °C for 20 s followed by 40 cycles of 94 °C for 30 s, 58 °C for 30 s and 72 °C for 10 s.

**Table 3:** Primer sequences for qPCR.

Gene ID^a^	Primer sequence (5’ to 3’)	Exon spanning

AAE3_02268	AGATGCGTATTCTGATGGTTGGTC	yes
	CCCACACTGTGAATGAGATGTTC	yes

AAE3_07669	ATTCCTACGATCCTTTTGCCG	yes
	GATCATATTGTTTCGGGAGTCCT	yes

AAE3_04120	GCGAAGACACAGTTTTGACAG	yes
	TGAGCTAACGTCATCAATCCC	yes

AAE3_10454	ATGCGACTTCAATCTTTTGGC	yes
	ATGTCGGCCATGCGTCTT	no

^a^Referring to the gene IDs from the genomic sequence of *C. aegerita* AAE-3 (http://www.thines-lab.senckenberg.de/agrocybe_genome).

## Supporting Information

File 1 ^1^H and ^13^C NMR spectra of compound **1** and ^1^H, ^13^C, COSY, ROESY, HSQC and HMBC NMR spectra of compounds **2**–**4**.
