# Supplementary material for: New terpenoids from the fermentation broth of the edible mushroom Cyclocybe aegerita
Source: Beilstein J Org Chem. 2019 Apr 30;15:1000–7. doi: 10.3762/bjoc.15.98 (PMC6541320; doi:10.3762/bjoc.15.98)
Supplement: File 1 — 1H and 13C NMR spectra of compound 1 and 1H, 13C, COSY, ROESY, HSQC and HMBC NMR spectra of compounds 2–4. [file Beilstein_J_Org_Chem-15-1000-s001.pdf]

BEILSTEIN JOURNAL OF ORGANIC CHEMISTRY

# Supporting Information

for

# New terpenoids from the fermentation broth of the edible mushroom *Cyclocybe aegerita*

Frank Surup, Florian Hennicke, Nadine Sella, Maria Stroot, Steffen Bernecker, Sebastian Pfütze, Marc Stadler and Martin Rühl

*Beilstein J. Org. Chem.* **2019,** *15,* 1000–1007. doi:10.3762/bjoc.15.98

## $^{1}H$ and $^{13}C$ NMR spectra of compound 1 and $^{1}H$, $^{13}C$, COSY, ROESY, HSQC and HMBC NMR spectra of compounds 2–4

License and Terms: This is a supporting information file under the terms of the Creative Commons Attribution License (http://creativecommons.org/licenses/by/4.0). Please note that the reuse, redistribution and reproduction in particular requires that the authors and source are credited. The license is subject to the *Beilstein Journal of Organic Chemistry* terms and conditions: (https://www.beilstein-journals.org/bjoc)

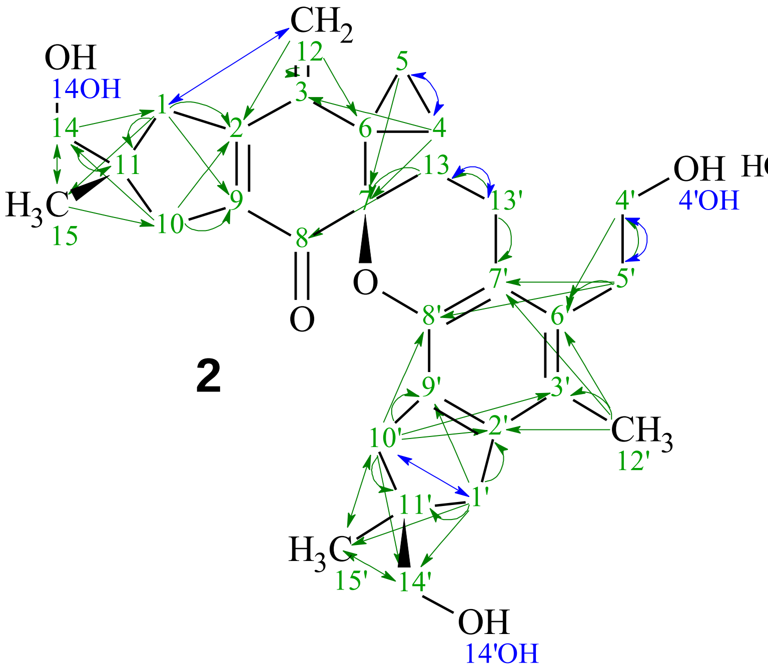

**2**

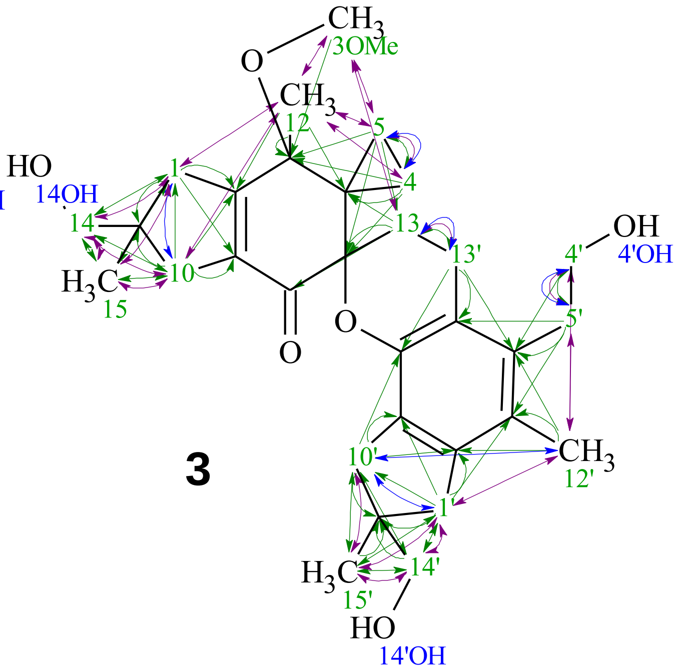

**3**

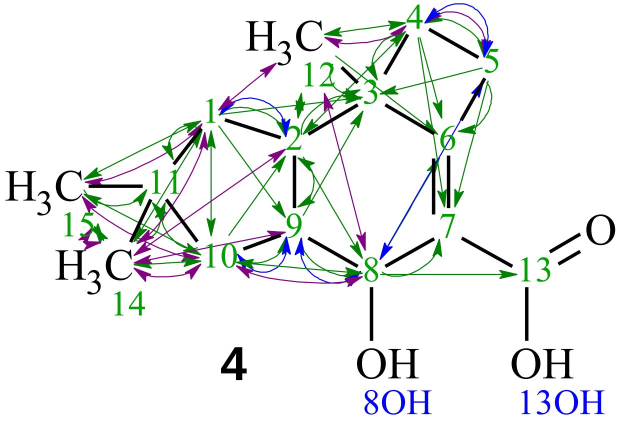

**4**

Figure S1. COSY (blue arrows), HMBC (green arrows) and ROESY (violet arrows) correlations indicating the structures of **2**–**4**.

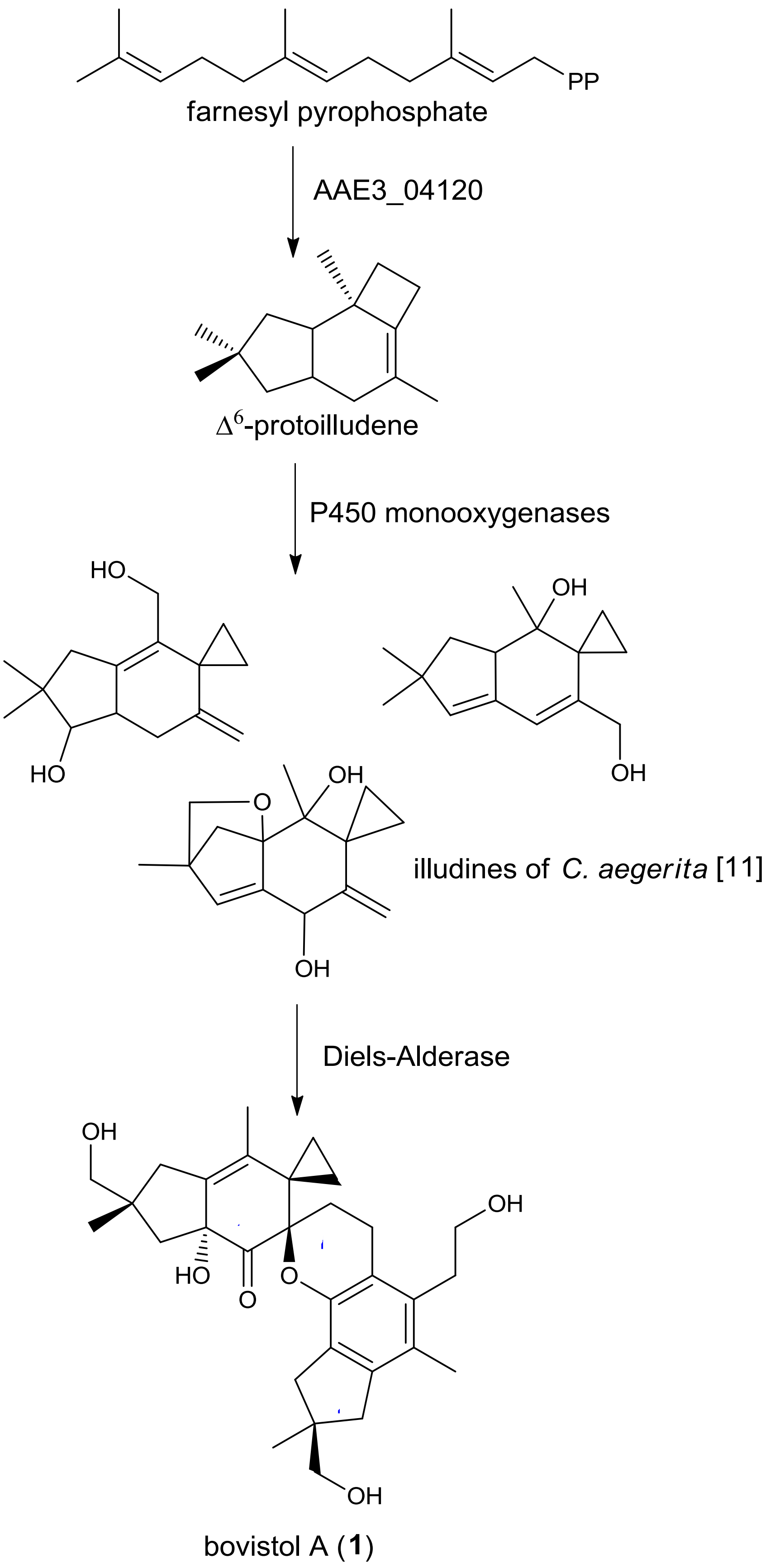


Figure S2. Proposed pathway for production of **1** in *C. aegerita*.

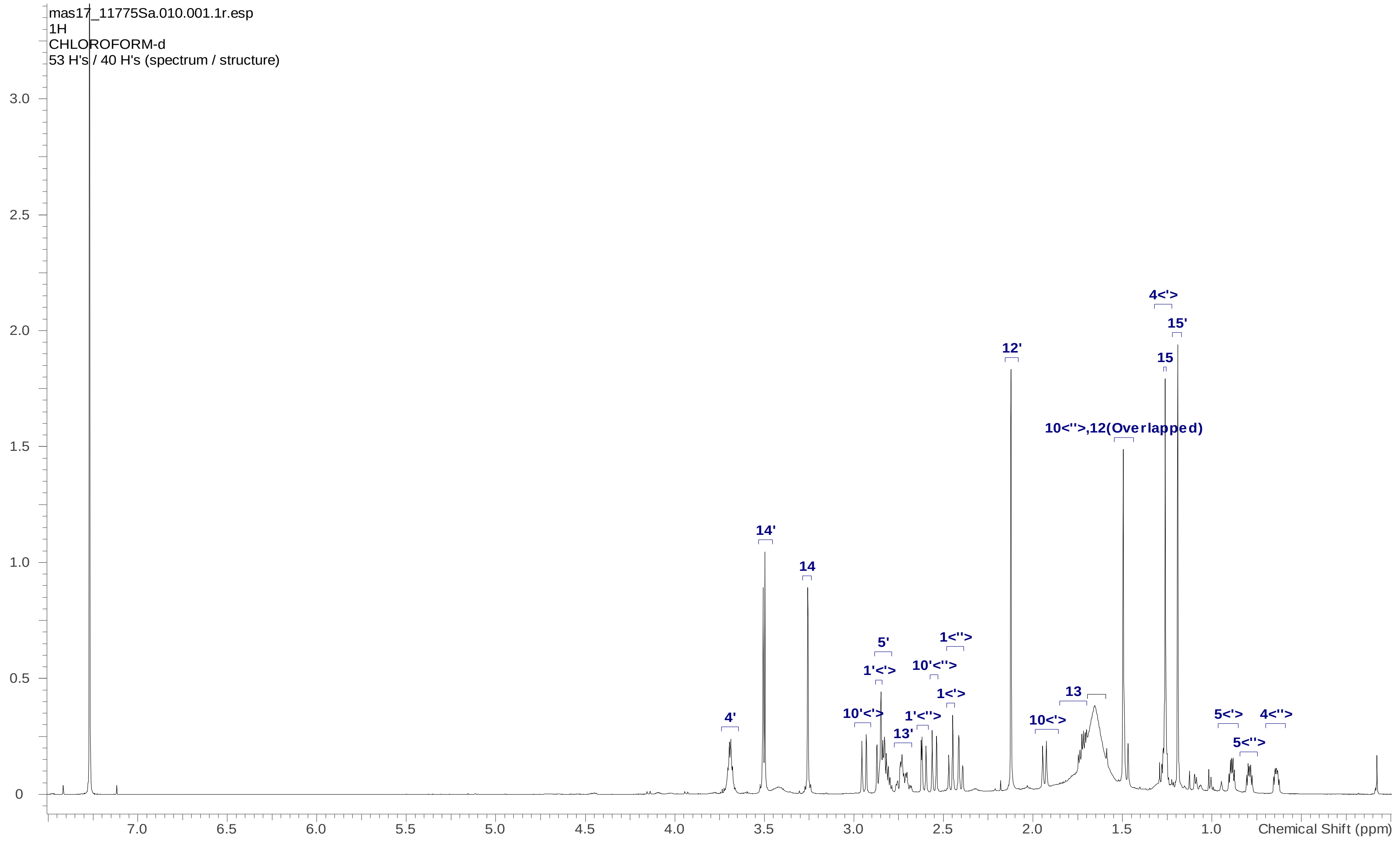


$^{1}$H NMR spectrum of **1** (700 MHz, $CDCl_3$).

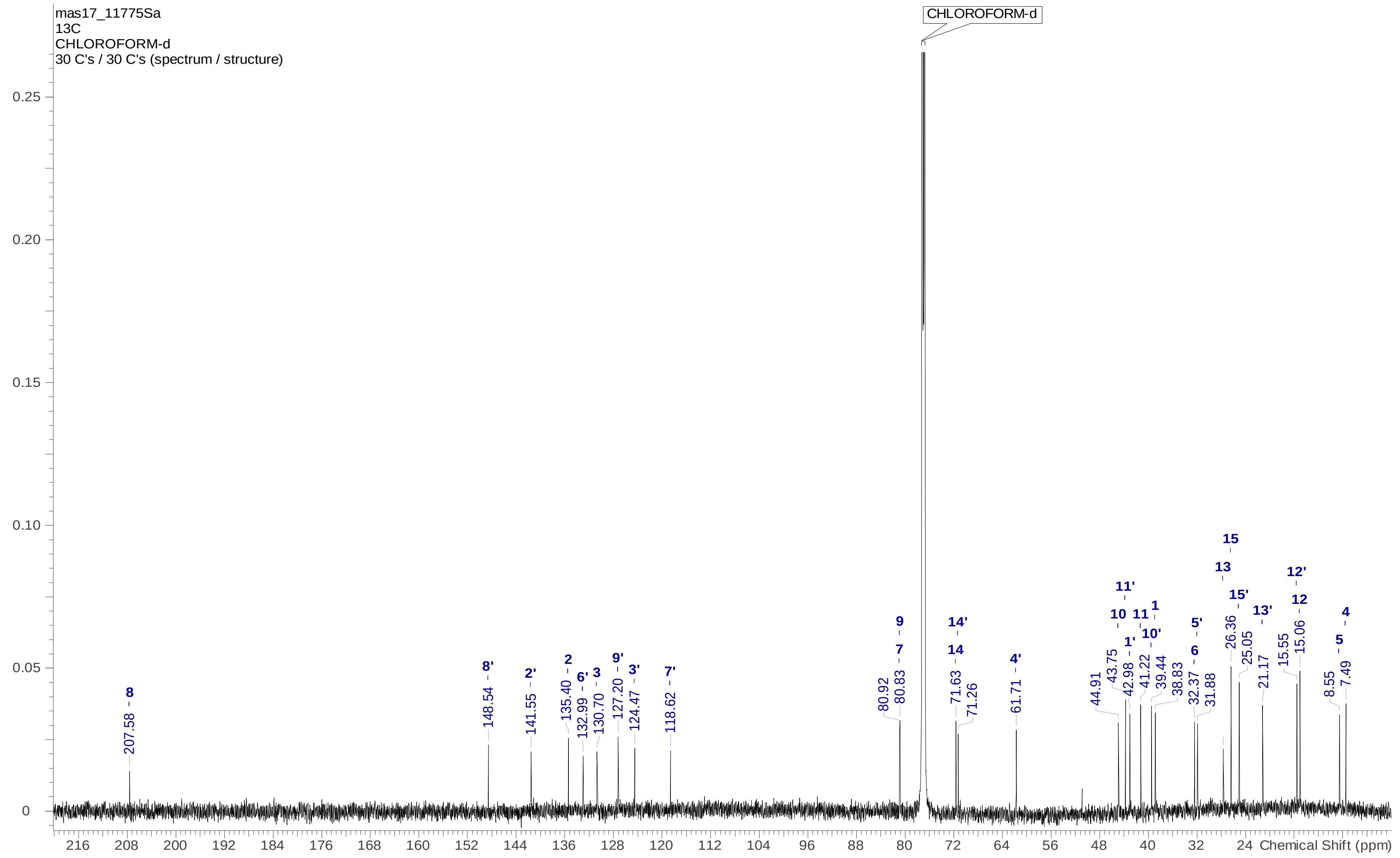


$^{13}$C NMR spectrum of **1** (175 MHz, $CDCl_3$).

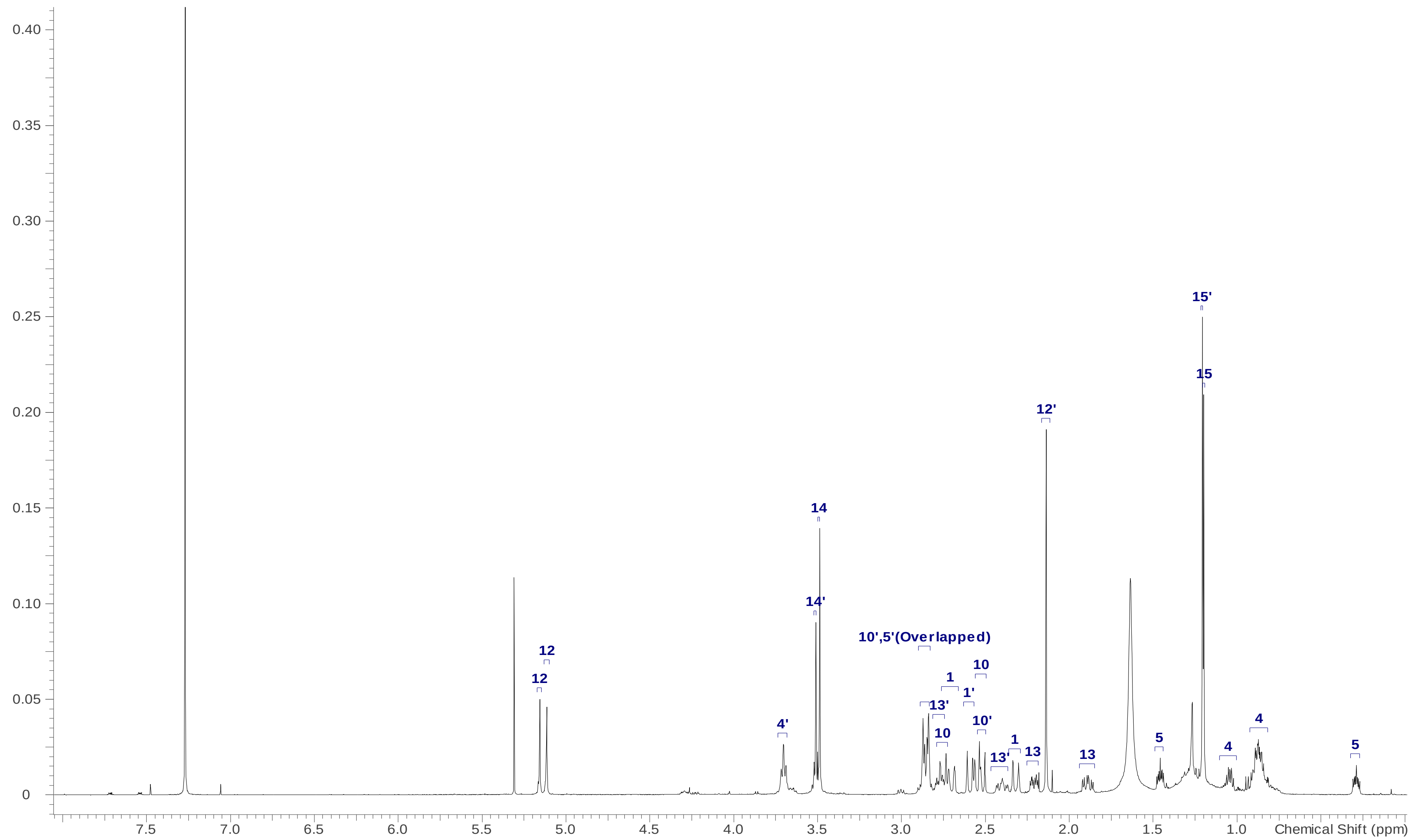


$^{1}$H NMR spectrum of **2** (700 MHz, $CDCl_3$).

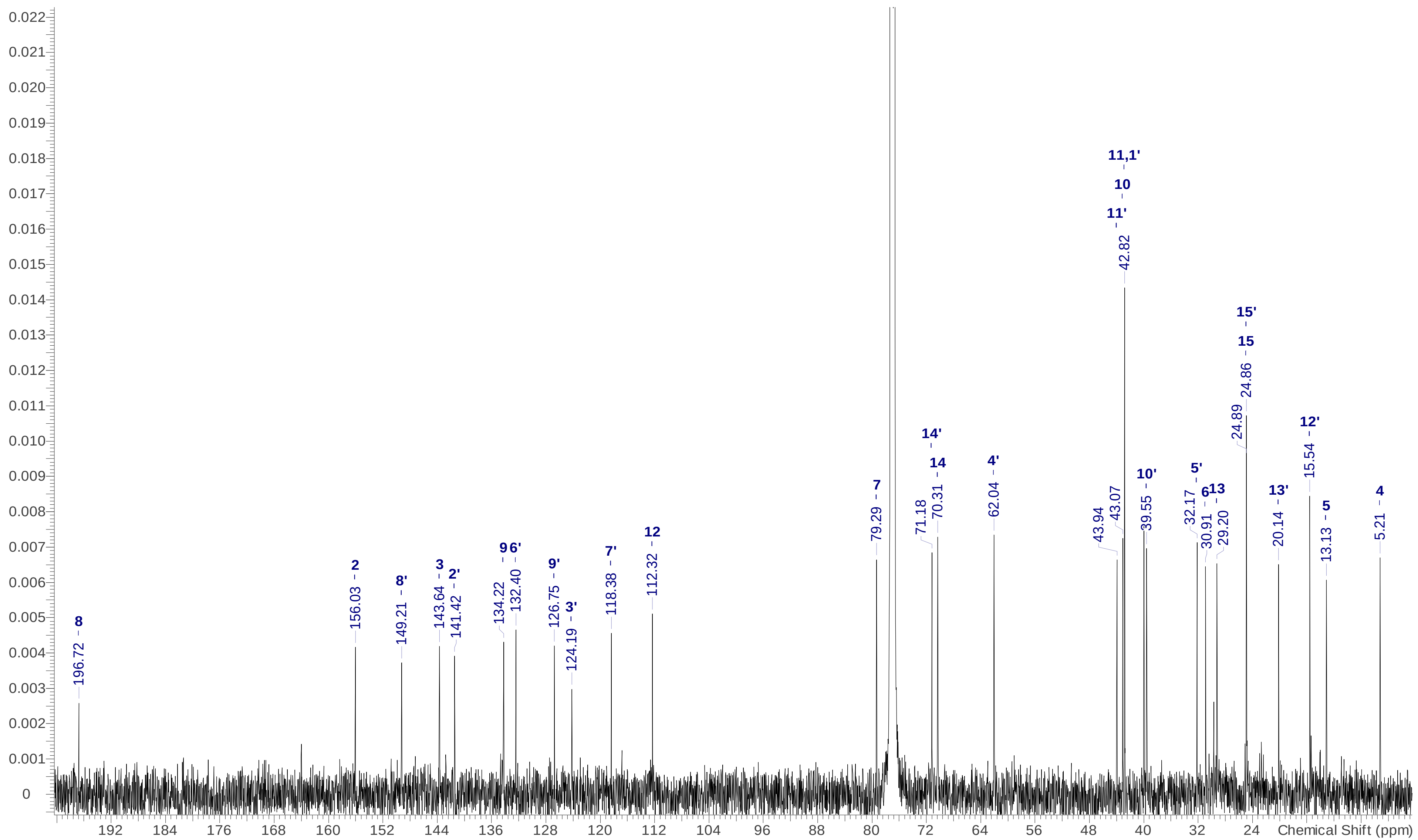


$^{13}C$ NMR spectrum of **2** (175 MHz, $CDCl_3$).

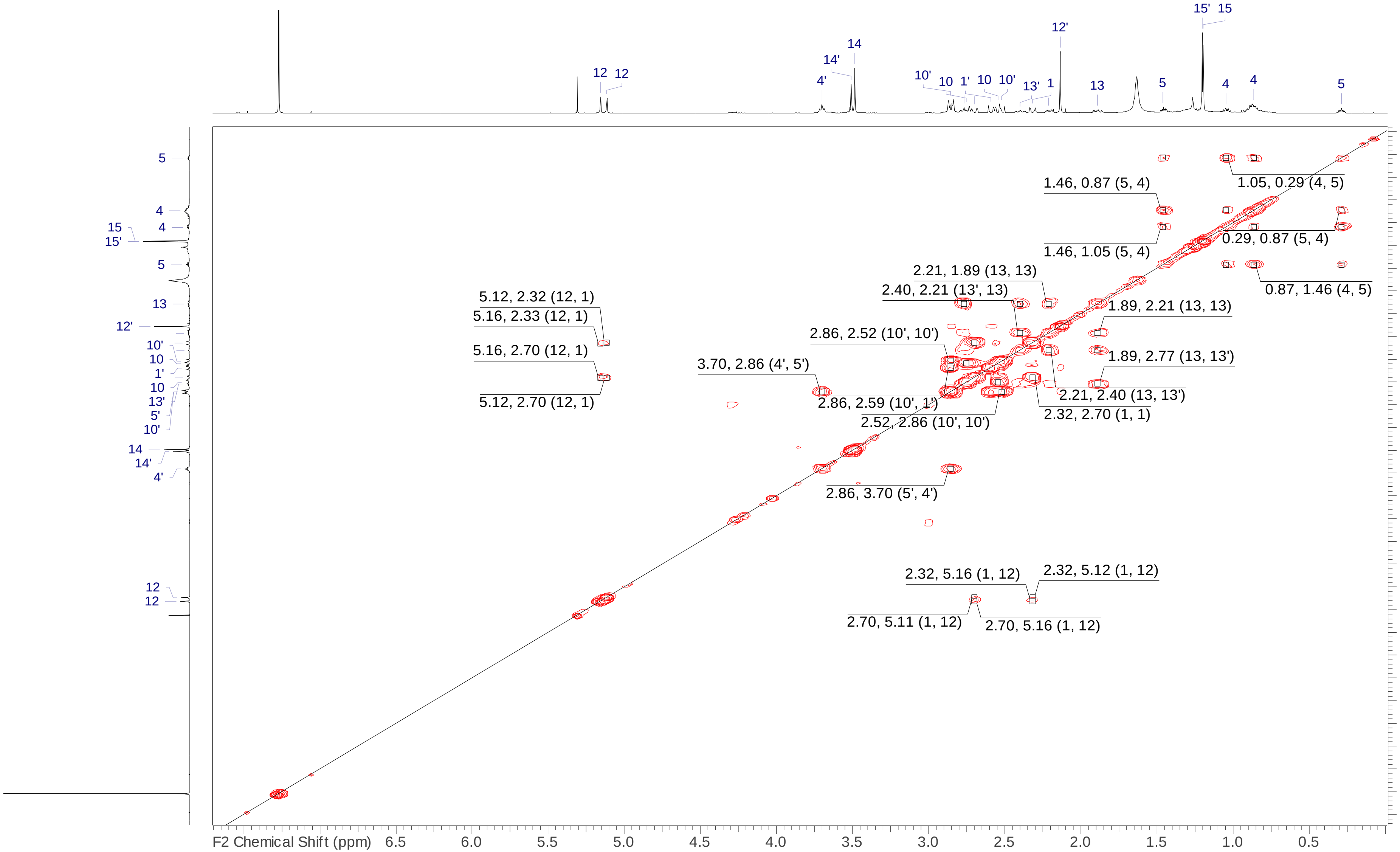


COSY NMR spectrum of **2** (700 MHz, $CDCl_3$).

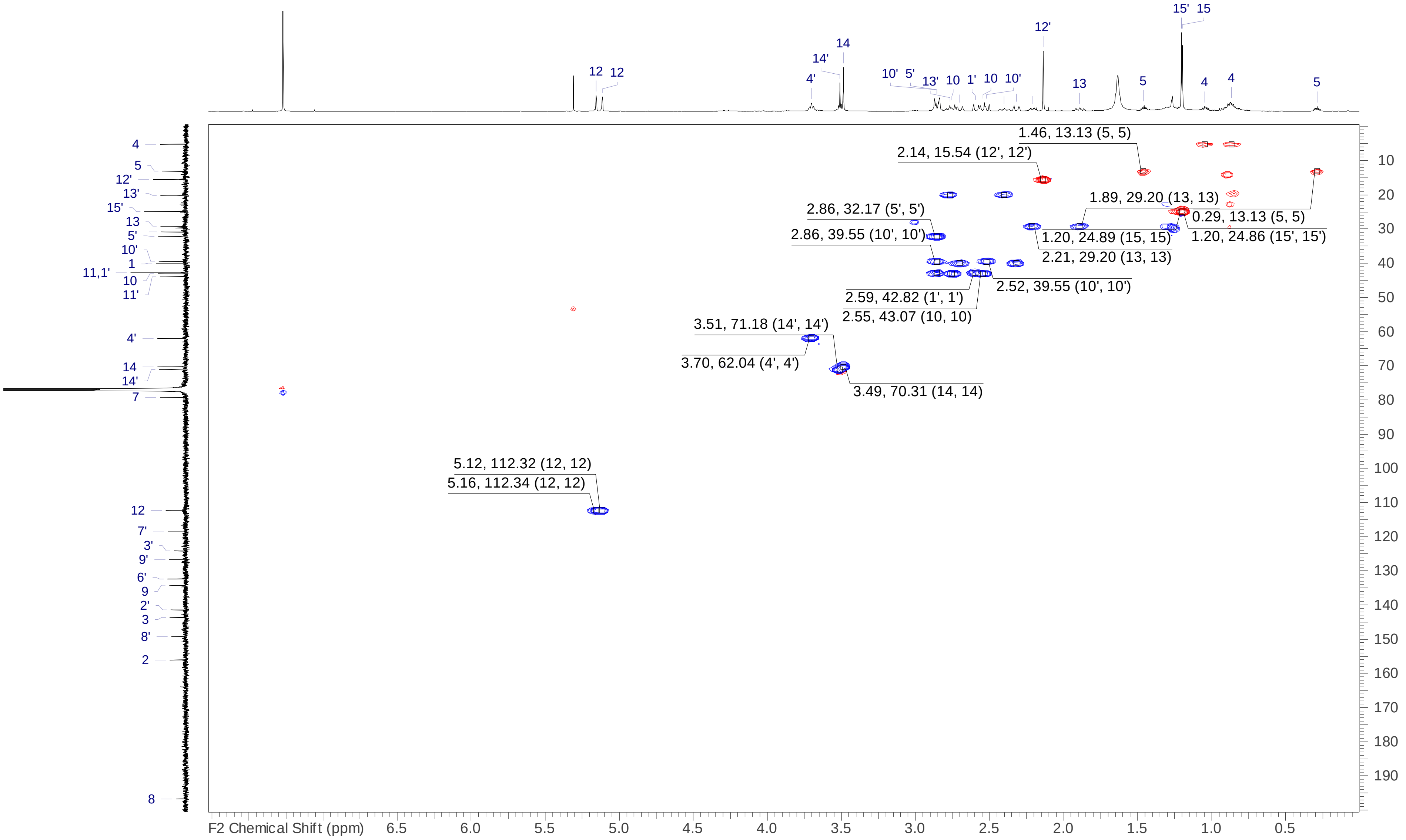


HSQC NMR spectrum of **2** (700 MHz, $CDCl_3$).

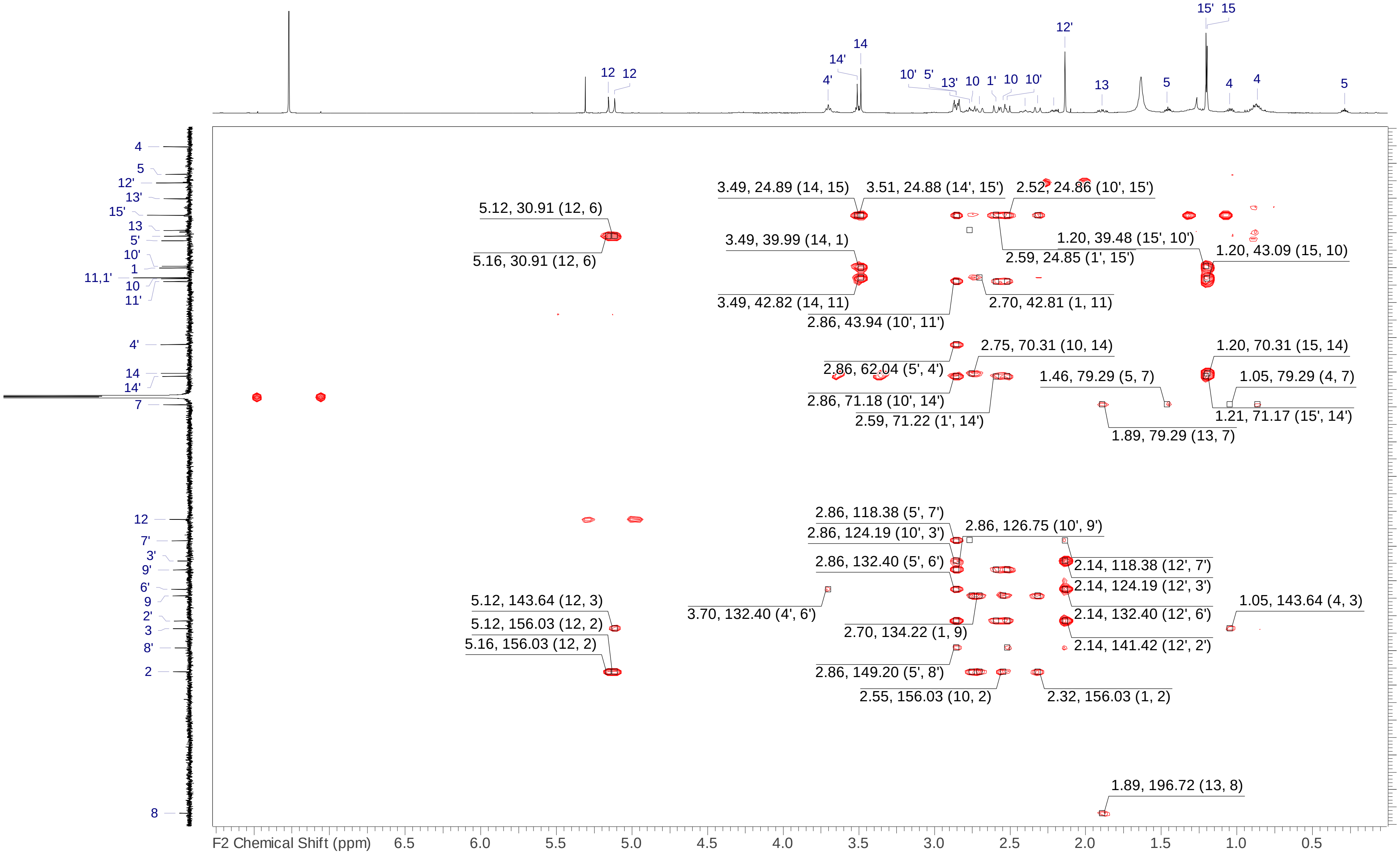


HMBC NMR spectrum of **2** (700 MHz, $CDCl_3$).

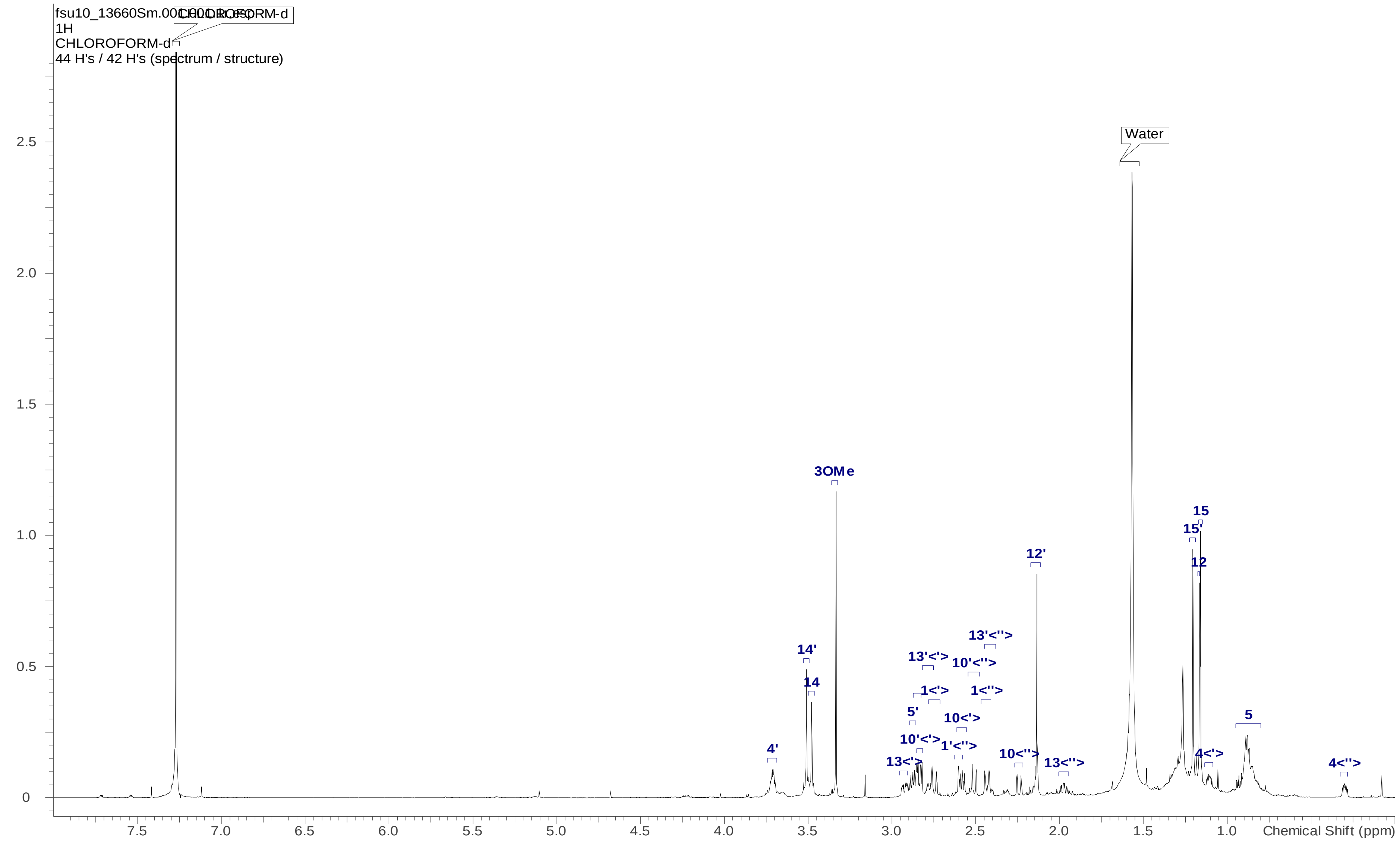


$^{1}$H NMR spectrum of **3** (700 MHz, $CDCl_3$).

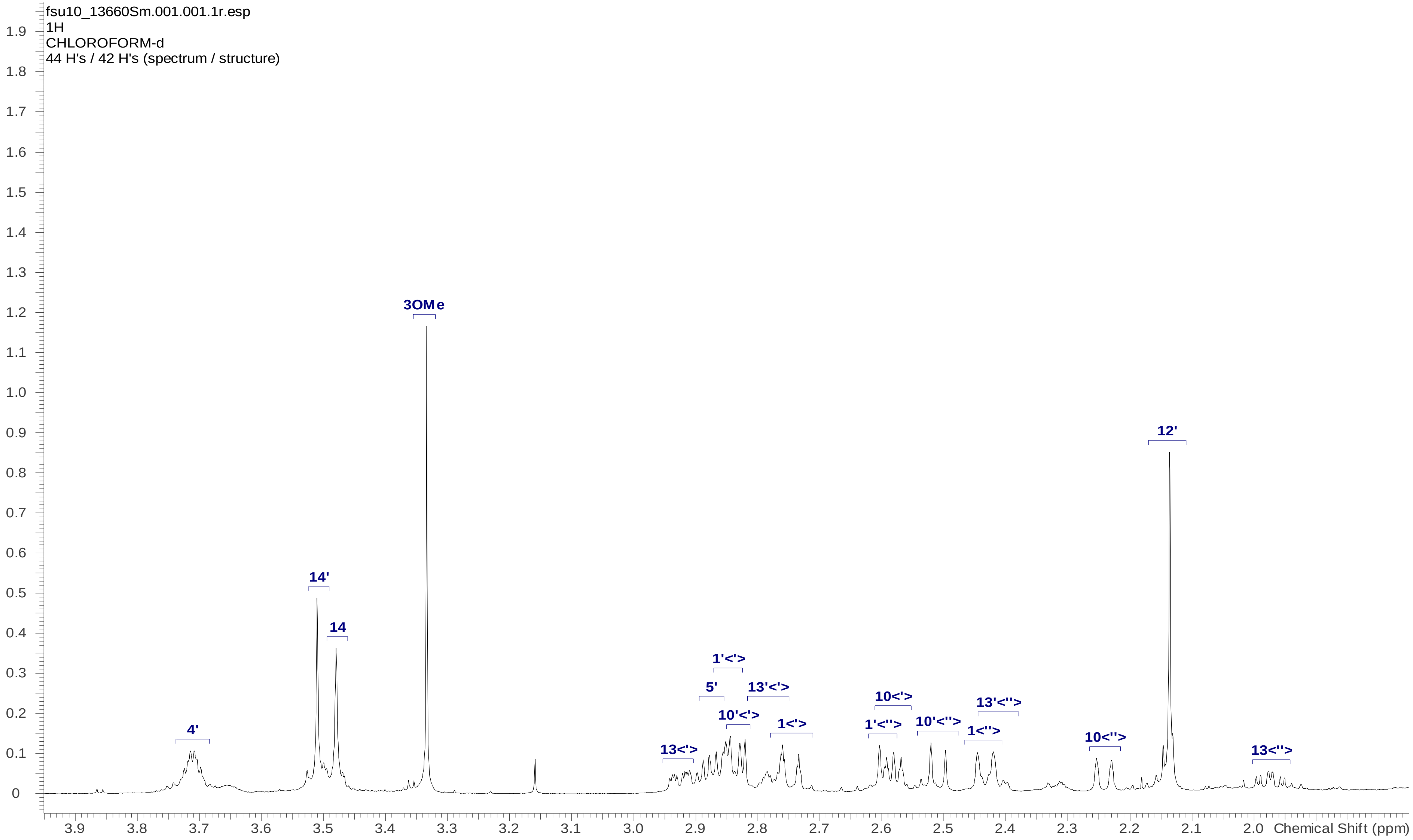


Section from $^{1}$H NMR spectrum of **3** (700 MHz, $CDCl_3$).

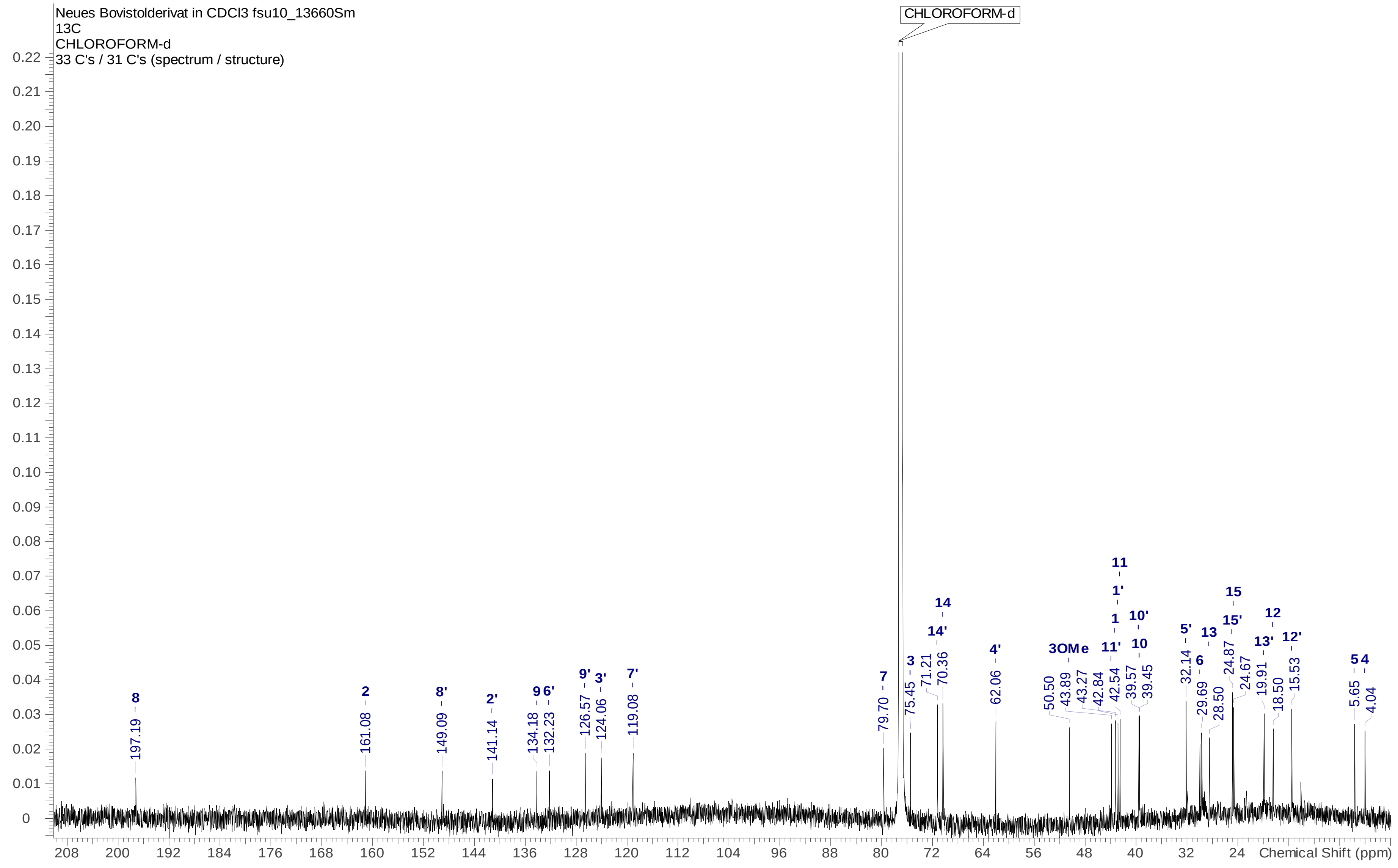


$^{13}$C NMR spectrum of **3** (175 MHz, $CDCl_3$).

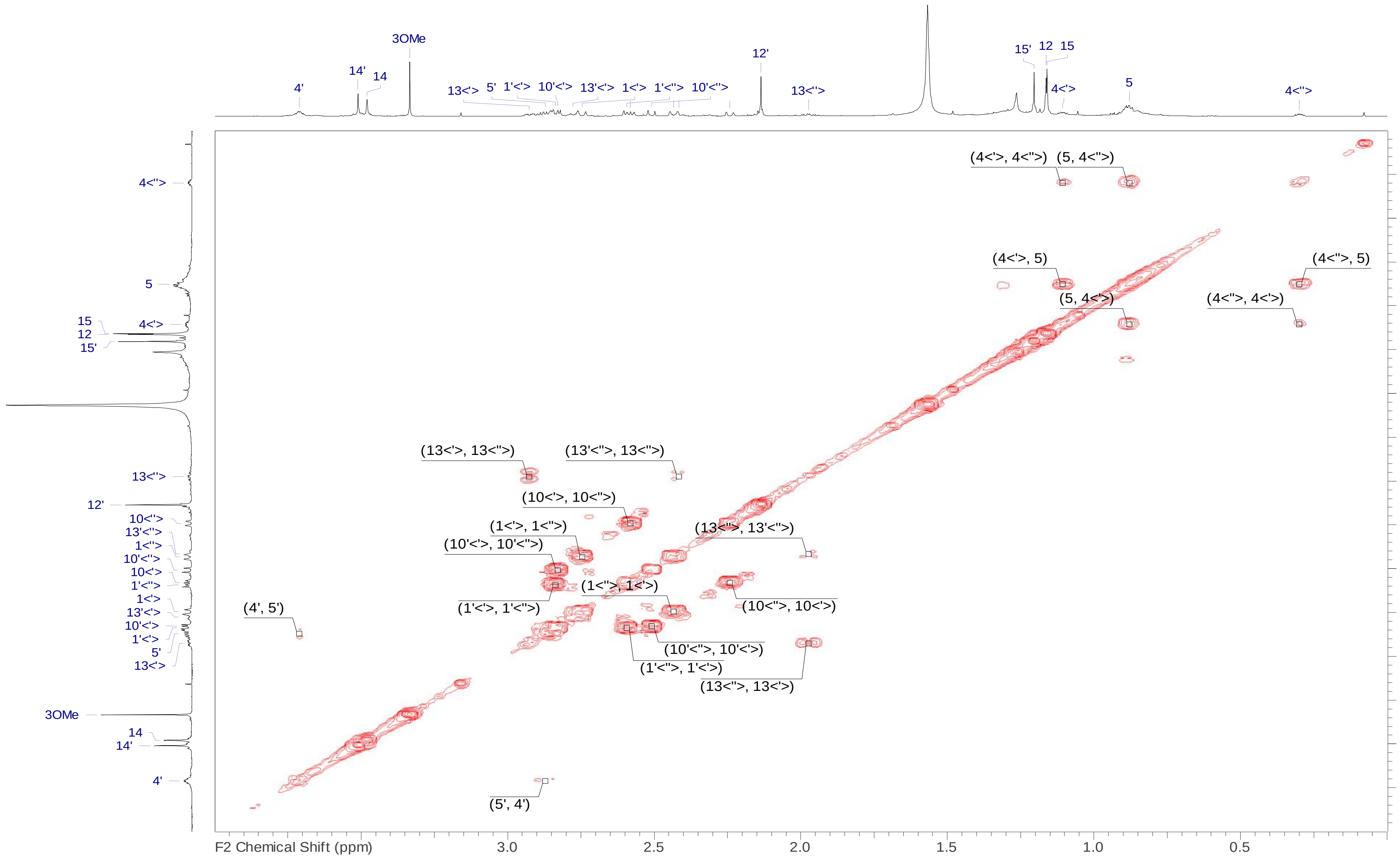


COSY NMR spectrum of **3** (700 MHz, $CDCl_3$).

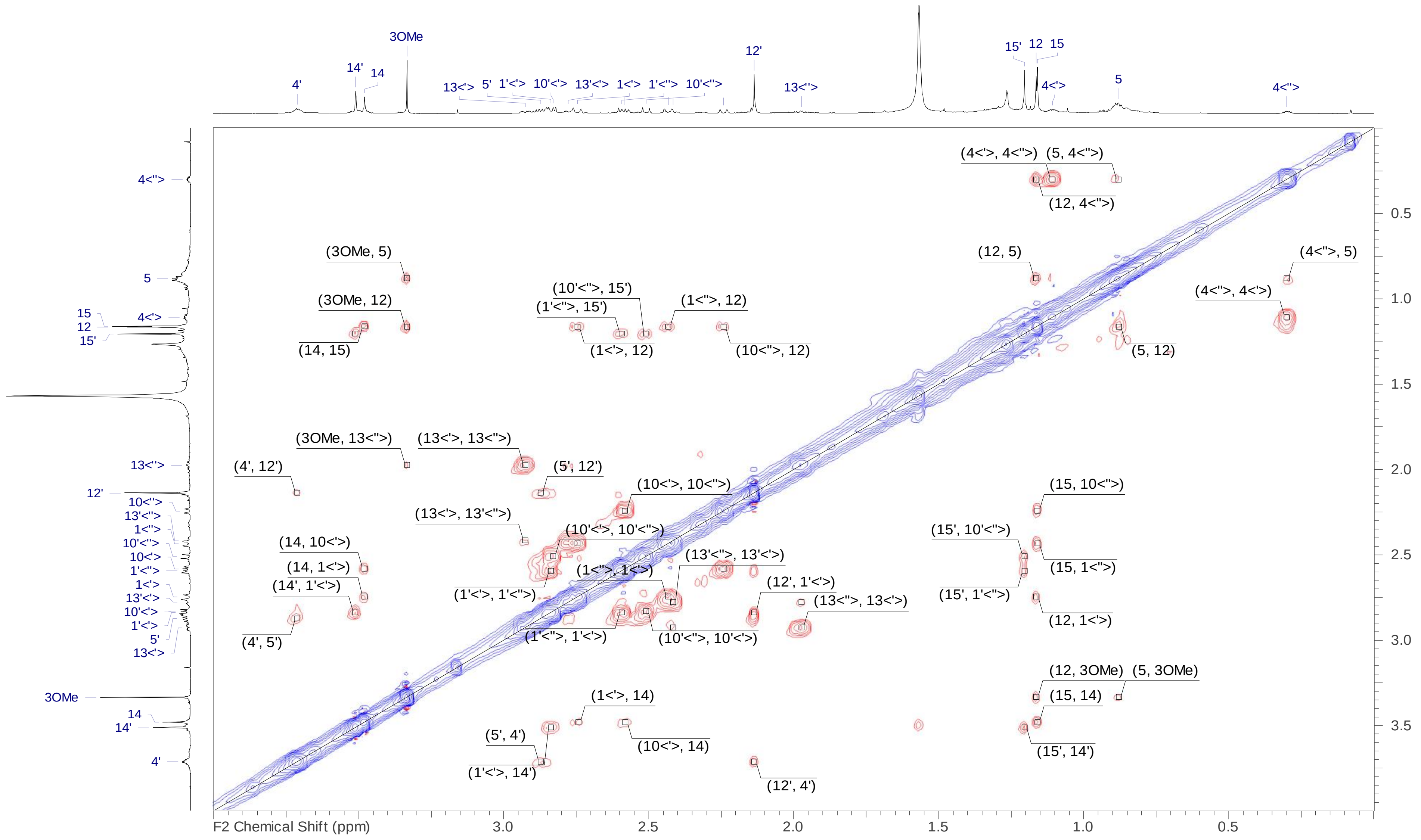


ROESY NMR spectrum of **3** (700 MHz, $CDCl_3$).

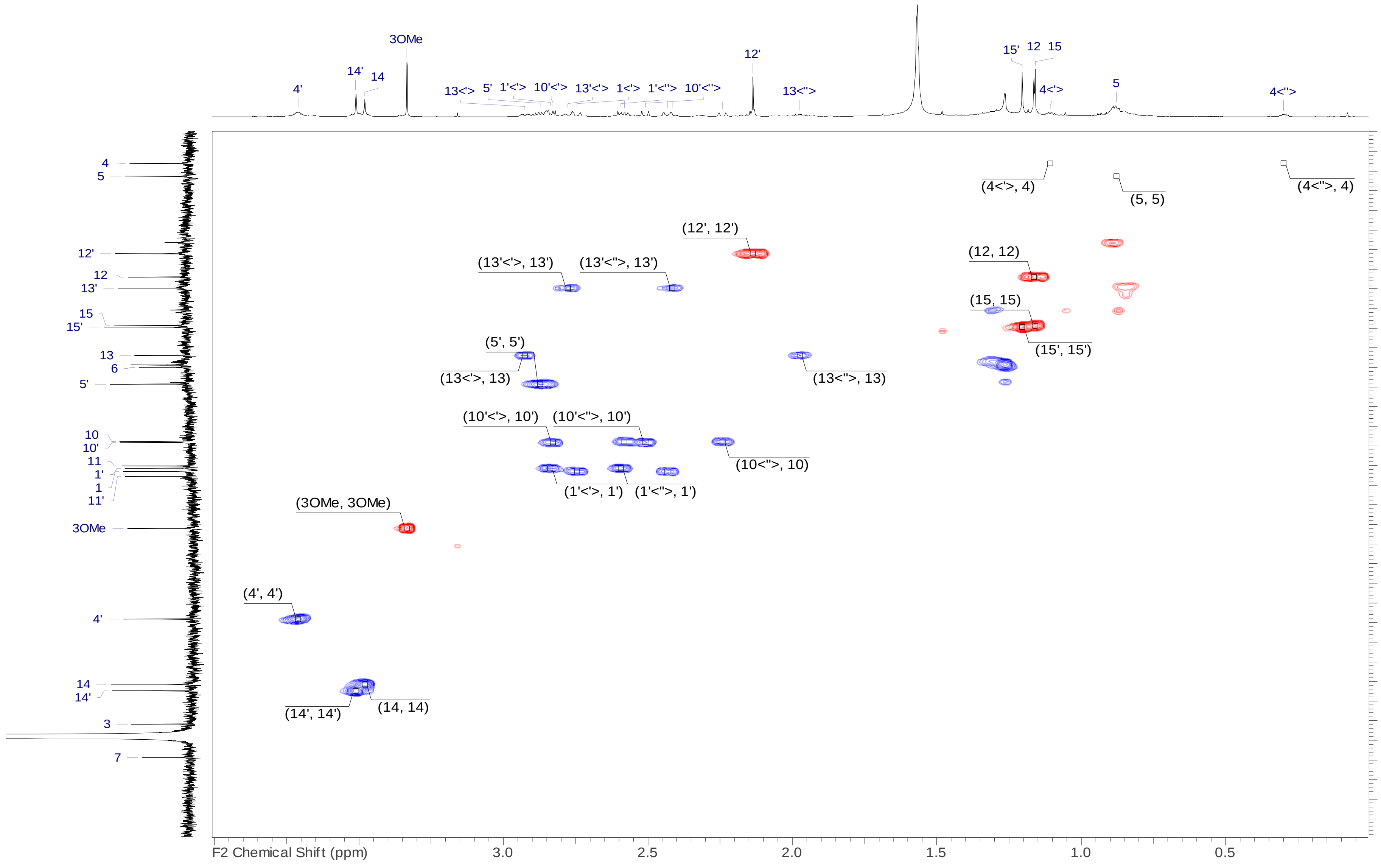


ROESY NMR spectrum of **3** (700 MHz, $CDCl_3$).

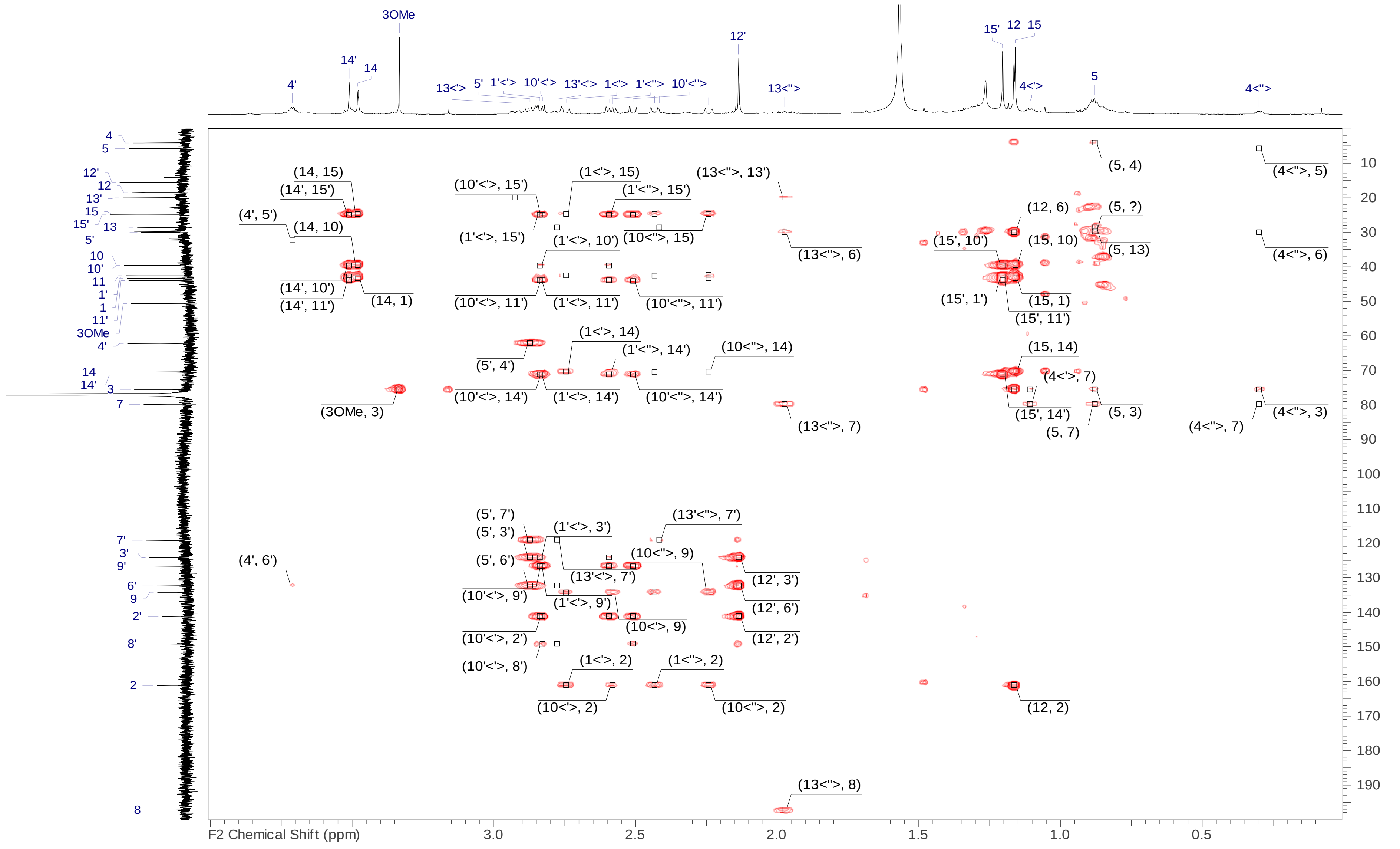


HMBC NMR spectrum of **3** (700 MHz, $CDCl_3$).

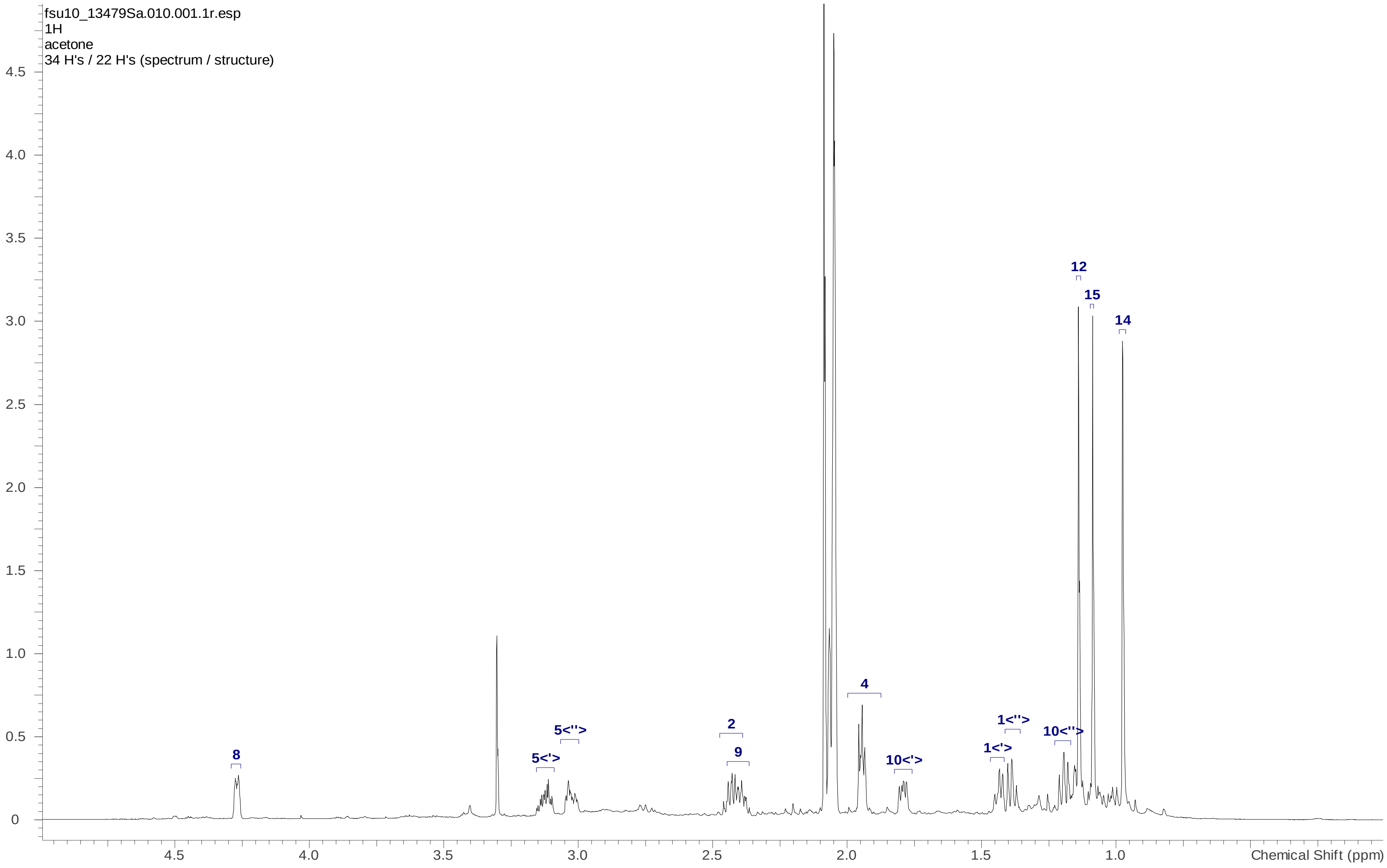


$^{1}$H NMR spectrum of **4** (700 MHz, acetone-$d_6$).

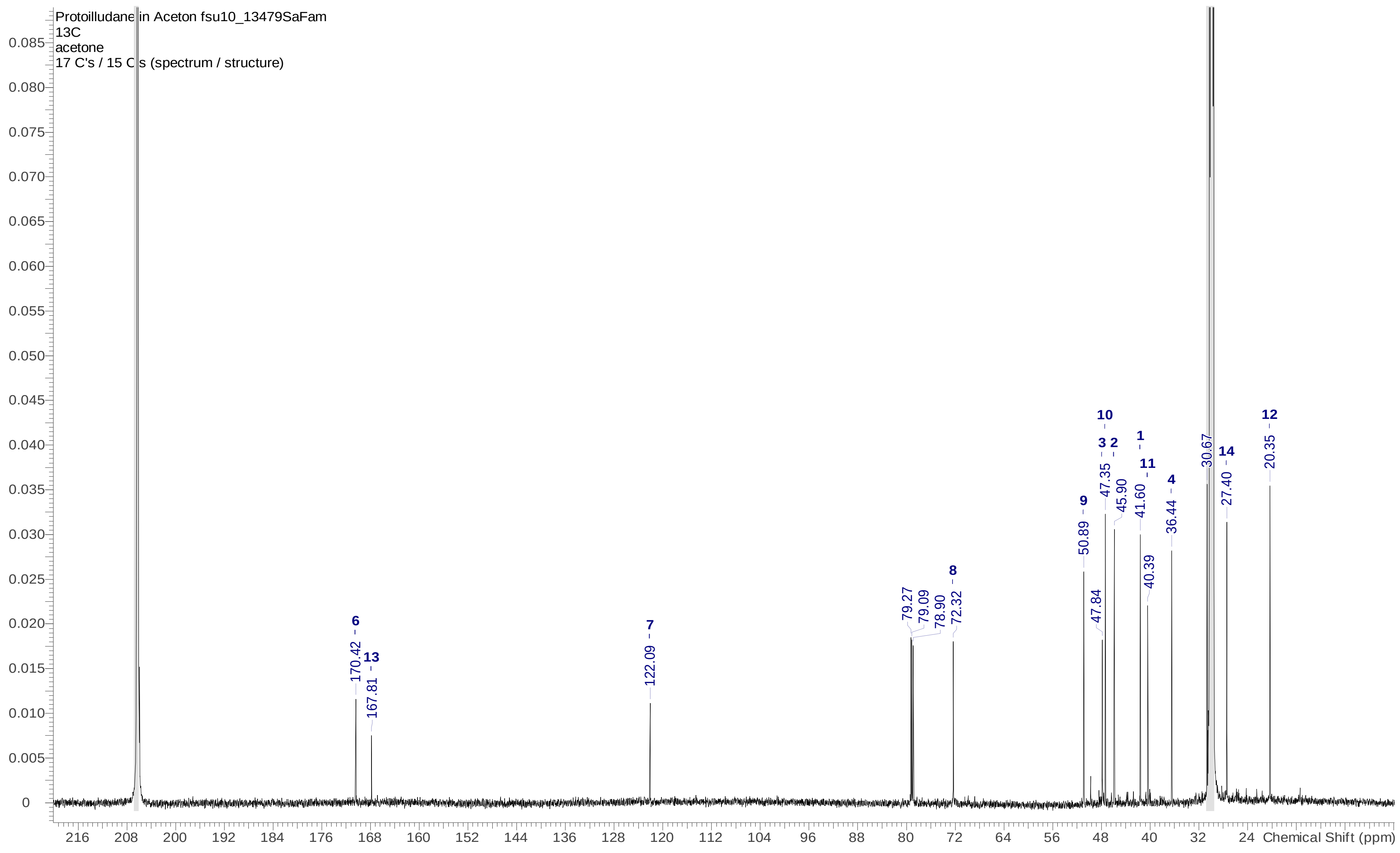


$^{13}$C NMR spectrum of **4** (175 MHz, acetone-$d_6$).

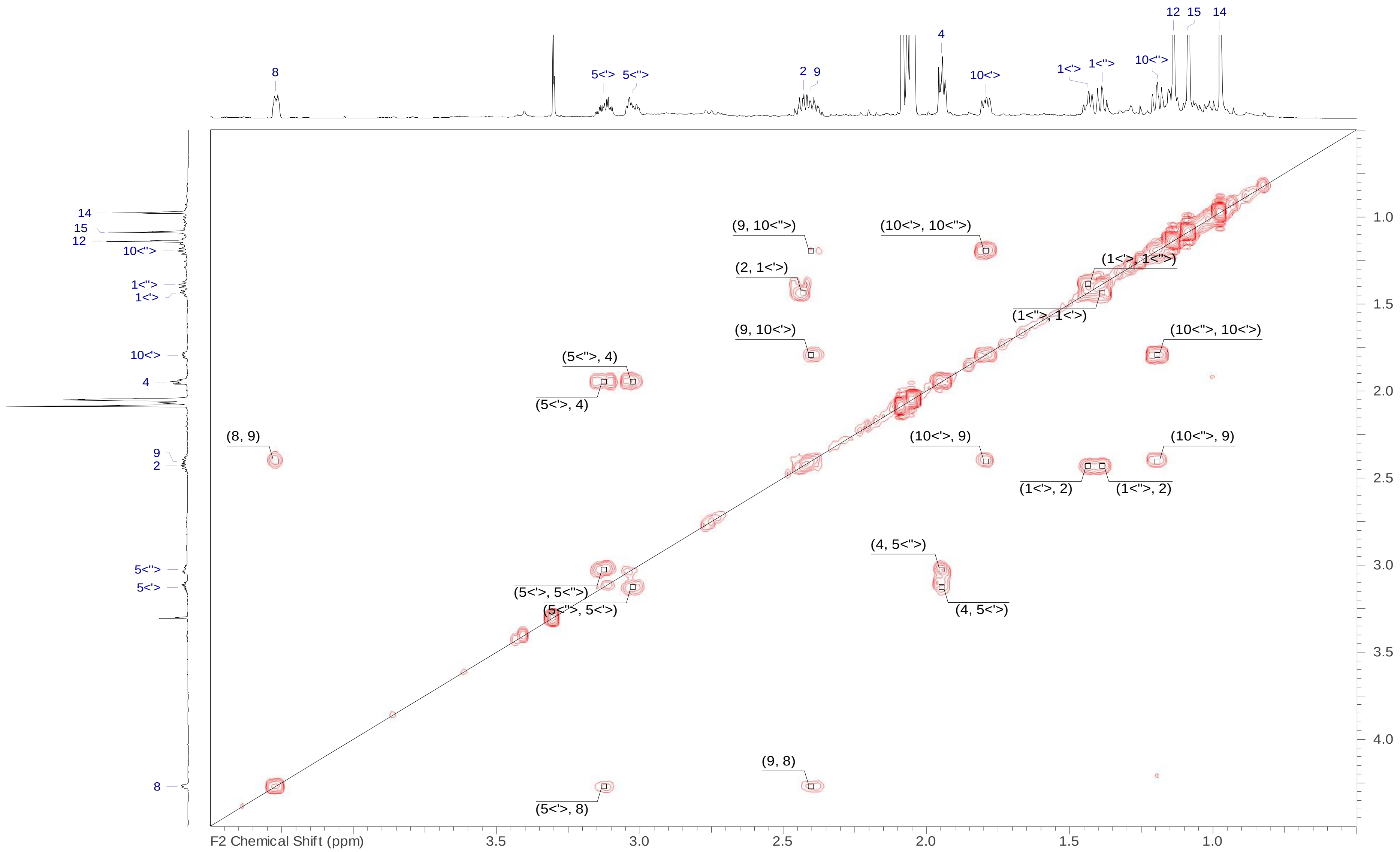


COSY NMR spectrum of **4** (700 MHz, acetone-$d_6$).

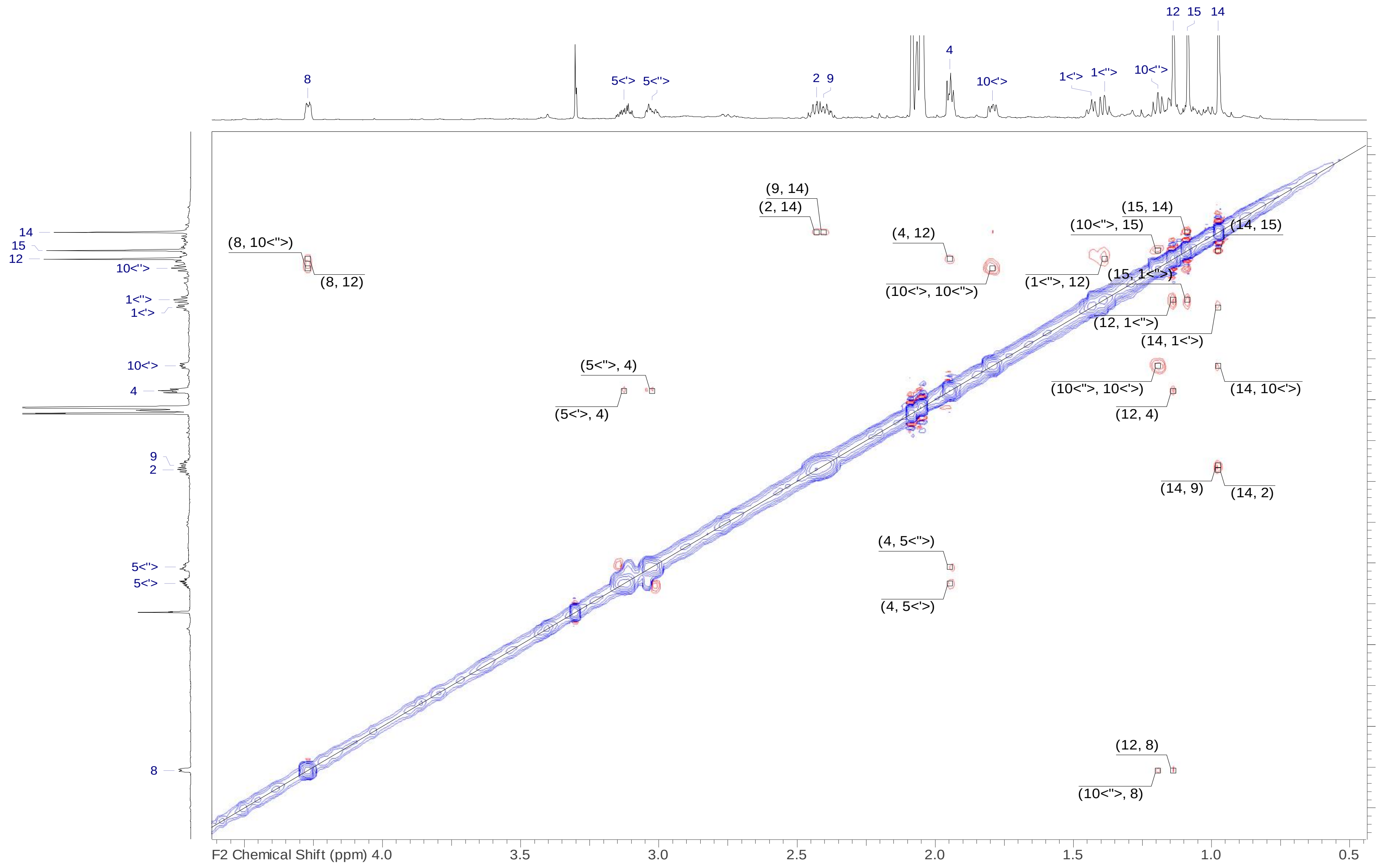


ROESY NMR spectrum of **4** (700 MHz, acetone-$d_6$).

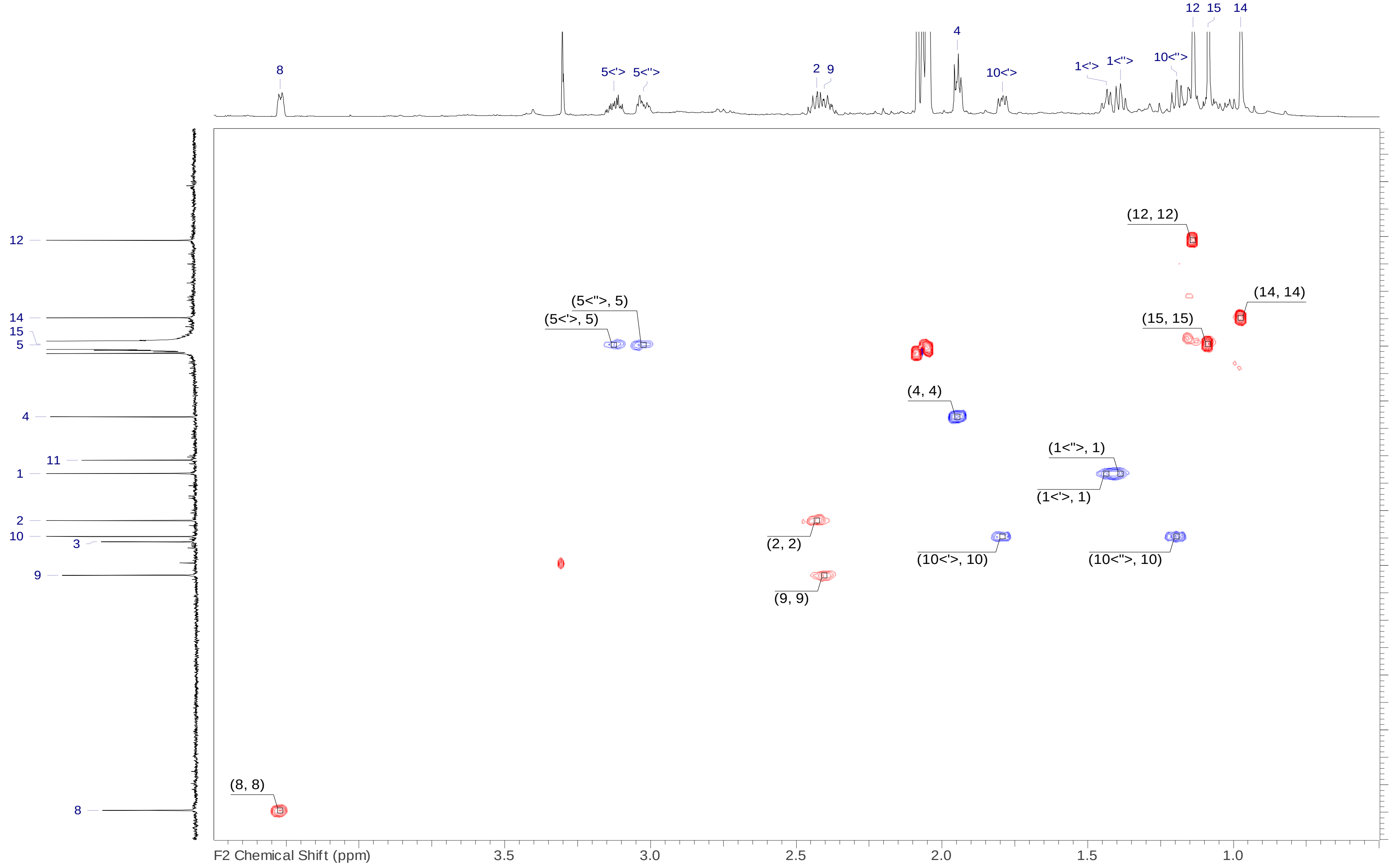


HSQC NMR spectrum of **4** (700 MHz, acetone-$d_6$).

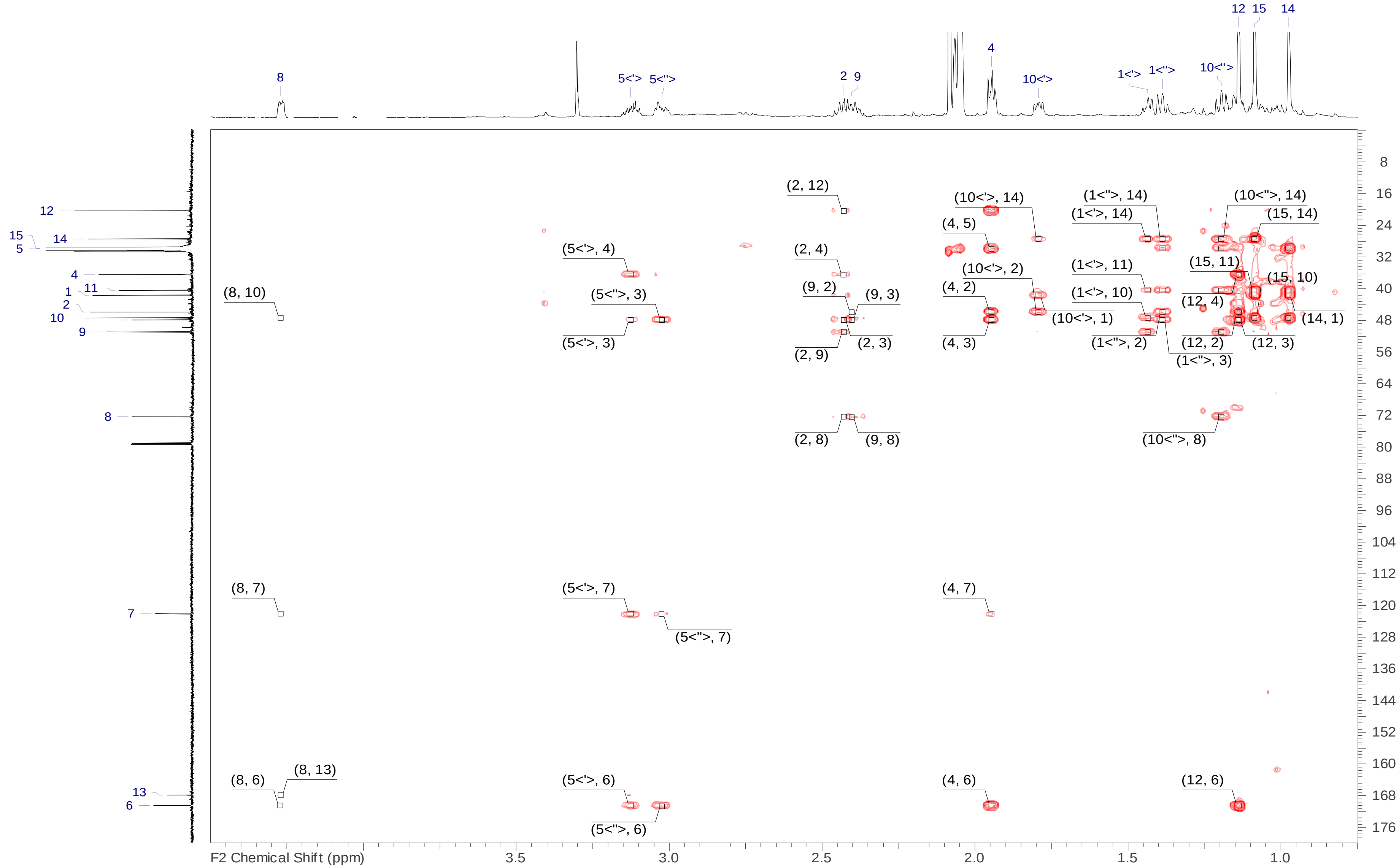

HMBC NMR spectrum of **4** (700 MHz, acetone-$d_6$).
